# Comparative analysis of advanced constraint-handling in quantum PSO with differential mutation for optimal power flow

**DOI:** 10.1038/s41598-026-46233-2

**Published:** 2026-05-21

**Authors:** Mourad Naidji, Alla Eddine Toubal Maamar, Mohamed Ilyas Rahal, Saad Mekhilef, Mehdi Seyedmahmoudian, Alex Stojcevski

**Affiliations:** 1 Laboratory of Electrical Engineering (LGE), Department of Electrical Engineering, University of M’Sila, P.O. Box 166 Ichebilia, M’Sila, 28000 Algeria; 2 Laboratoire Ingénierie des Systèmes et Télécommunications (LIST), Department of Electrical Systems Engineering, Faculty of Technology, University M’hamed Bougara of Boumerdes, Boumerdes, 35000 Algeria; 3 Laboratory of Automation and Signals of Annaba (LASA), Department of Electronics, Badji Mokthar-Annaba University, P.O. Box 12, Annaba, 23000 Algeria; 4 School of Engineering, Swinburne University of Technology, Hawthorn, Vic 3122 Australia; 5 Curtin Singapore, Curtin University, Singapore, Singapore

**Keywords:** Optimal power flow (OPF), QPSODM algorithm, Constraint handling (CH), Epsilon (ε) constraint (ECO), Superiority of feasible solutions (SFS), Stochastic ranking (SRA), Energy science and technology, Engineering, Mathematics and computing

## Abstract

Optimal Power Flow (OPF) is a highly nonlinear and constrained optimization problem that seeks optimal operating conditions while ensuring secure and efficient power system operation. Although metaheuristic algorithms have demonstrated strong global search capability for OPF, their performance is often limited by ineffective constraint handling. This paper presents a systematic investigation of three advanced constraint-handling (CH) techniques, Epsilon (ε) constraint (ECO), Superiority of Feasible Solutions (SFS), and Stochastic Ranking (SRA), when integrated into a unified Quantum-behaved Particle Swarm Optimization with Differential Mutation (QPSODM) framework. The proposed approaches are evaluated on IEEE 30, 57, and 118 bus test systems under multiple OPF objectives, including fuel cost, emission, voltage deviation, and power loss, while considering practical modeling features such as valve-point loading and multi-fuel generation. Statistical significance is assessed using the Wilcoxon signed-rank test complemented by effect size analysis. Numerical results indicate that QPSODM–ECO consistently achieves superior feasibility and convergence behavior, yielding up to 3.8% cost reduction and significantly lower constraint violations compared to SFS and SRA in large-scale systems. SFS exhibits comparable performance in several cases, whereas SRA shows inferior robustness under tight constraints. These findings confirm that the ε-constraint strategy is particularly well suited to quantum-behaved swarm dynamics and highlight the critical role of constraint-handling mechanisms in advanced OPF solvers.

## Introduction

### Background and motivation

Optimal Power Flow (OPF) is a critical and challenging problem in modern power systems, seeking to determine the optimal operating settings of unit generators while satisfying all operational and technical constraints^1^. OPF problems typically aim to minimize generation cost, power losses, emissions, or improve voltage stability while ensuring system security and reliability. However, due to their non-linear, non-convex^2,3^, and highly constrained nature, OPF formulations remain difficult to solve using classical optimization techniques. Traditional deterministic approaches, such as Newton-based and interior-point methods^4^, often suffer from convergence difficulties and entrapment in local optima, particularly when practical modeling features such as valve-point effects, prohibited operating zones, and multi-fuel generation are considered. Consequently, metaheuristic optimization algorithms have gained increasing attention due to their global search capability and flexibility in handling diverse OPF objectives^5^.

### Related work and literature review

A wide range of evolutionary and swarm-based algorithms have been proposed for solving OPF problems, including backtracking search algorithm (BSA)^6^, improved colliding bodies optimization (ICBO)^7^, slime mould algorithm (SMA)^8^, enhanced performance-based differential search algorithm (EPDSA)^9^, and catch fish optimization algorithm (CFOA)^10^. Numerous hybrid and improved metaheuristics have also been explored, such as non-dominated sorting colliding bodies optimization (NSCBO)^11^, quasi-oppositional sine–cosine algorithm (QOSCA)^12^, artificial rabbits optimization (ARO)^13^, white shark optimizer (WSO)^14^, and ameliorated moth swarm algorithm (AMSA)^15^. In parallel, machine learning and deep reinforcement learning approaches have emerged, including convex-constrained soft actor–critic methods^16^ and hybrid ML-based optimization strategies^17^. Mathematical and decomposition-based approaches have also contributed to OPF advancements through dynamic security constraints^18^, neurodynamic models^19^, branch-and-bound frameworks^20^, and proximal optimization techniques^21^. Despite these advances, no single method has proven universally effective across all OPF formulations and operating conditions.

### Constraint handling in OPF optimization

Constraint handling (CH) plays a pivotal role in metaheuristic-based OPF solvers, as feasibility and optimality must be achieved simultaneously. While traditional penalty function approaches have long dominated OPF studies, more advanced CH techniques have recently emerged. These include superiority of feasible solutions (SFS), self-adaptive penalty (SAP), epsilon constraint (ECO), and hybrid strategies, which have been integrated with differential evolution, MOEA/D, and other metaheuristics^22–27^. Recent studies have also explored constraint handling within bio-inspired algorithms^24^, reinforcement learning frameworks^28^, integrating an eliminating strategy into NSGA-III^29^, stochastic OPF formulations^30^, constraint-relaxation techniques^31^, and chance-constrained optimization^32^. Although these approaches demonstrate promise, their application remains fragmented and often limited to a single CH mechanism within a specific optimizer.

### Research gap

Despite the growing body of literature on OPF optimization and constraint handling, a clear gap remains. Existing studies rarely isolate the effect of the constraint-handling strategy itself, as algorithmic modifications and CH mechanisms are often introduced simultaneously. As a result, the relative strengths and weaknesses of advanced CH techniques, such as ECO, SFS, and SRA, have not been systematically compared under identical optimization settings, OPF objectives, and realistic modeling conditions. This lack of fair and unified comparison limits the understanding of how CH strategies influence feasibility, convergence behavior, and robustness in highly constrained and non-convex OPF problems.

### Contributions of this work

Despite its simplicity and wide adoption, conventional particle swarm optimization (PSO) suffers from several limitations when applied to highly constrained and non-convex OPF problems, including premature convergence, loss of population diversity, and sensitivity to parameter tuning. These issues become more pronounced in realistic OPF formulations involving valve-point effects, multi-fuel cost functions, and large-scale networks. To alleviate these shortcomings, quantum-behaved PSO enhances global exploration through probabilistic search, while differential mutation further improves diversity preservation and convergence robustness.

Motivated by these challenges, this paper presents a unified comparative study of three advanced constraint-handling techniques, ECO, SFS, and SRA, embedded within a Quantum-behaved Particle Swarm Optimization with Differential Mutation (QPSODM) framework. By maintaining an identical core optimizer and varying only the constraint-handling strategy, a fair and transparent assessment of CH effectiveness is achieved.

The main contributions of this work are summarized as follows:

- Unified integration and comparison of ECO, SFS, and SRA under identical optimization settings.
- Inclusion of practical OPF modeling features such as valve-point loading and multi-fuel cost functions.
- Rigorous performance validation using non-parametric statistical tests.
- Extensive evaluation on IEEE benchmark systems, including the IEEE 118-bus system, demonstrating the scalability and effectiveness of the proposed approach for large-scale OPF problems in the context of highly constrained, non-linear formulations incorporating valve-point effects and multi-fuel cost modeling.

**Fig. 1:**
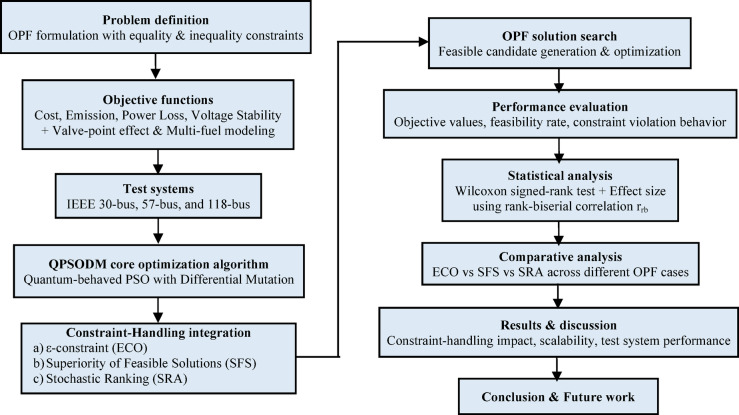
Overall workflow of the proposed QPSODM-based OPF framework with integrated constraint-handling techniques (ECO, SFS, and SRA).

To provide a clear overview of the proposed methodology, Fig. 1 illustrates a block diagram summarizing the overall workflow of the study, from OPF formulation and constraint handling to optimization and performance evaluation.

The remainder of this paper is organized as follows. Section 2 presents the analytical formulation of the OPF problem, including the objective functions considered in this study. Section 3 describes the integration of the proposed CH methods into the QPSODM algorithm. Section 4 discusses the case studies and reports the simulation results, performance comparisons, and statistical analyses. Finally, Section 5 concludes the paper and outlines key findings and directions for future research.

## Analytical model of optimal power flow

The OPF problem represents a highly intricate and non-linear optimization task characterized by its non-convex nature. Its primary goal is to determine the most efficient operating conditions of a power system by minimizing one or multiple objective functions while satisfying a set of equality and inequality constraints. The mathematical formulation of the OPF can be expressed as follows^22^:

1 $$\:Min\:F(x,u)$$

$$\:subject\:to:\:\left\{\begin{array}{c}H(x,u)=0\\\:G(x,u)\le\:0\end{array}\right.$$

where *x* represents the vector of control (independent) variables and *u* represents the vector of state (dependent) variables. The objective function is represented by *F(x*,* u)*, while *H(x*,* u)* corresponds to the set of equality constraints, and *G(x*,* u)* represents the set of inequality constraints.

### Control variables (independent)

The vector representing the set of variables that govern the power flow in the network is expressed as^22^:

2 $$\:x=[{Pg}_{2},\dots\:,{Pg}_{Ng};{Vg}_{1},\dots\:,{Vg}_{Ng};{T}_{1},\dots\:,{T}_{NT};{Qc}_{1},\dots\:,{Qc}_{Nc}]$$

where, *Pg*_i_ is the active power of a generator at the *i*^th^ bus (excluding the slack generator). The selection of the slack bus does not affect the formulation; any generator bus can be the slack bus. *Ng* represents the voltage magnitude at the *i*^th^ generator bus (PV), *T*_j_ is the Tap setting of the *j*^th^ branch transformer, and *Qc*_k_ represents the shunt capacitor at the *k*^th^ bus. In addition, *g*, *NT*, and *NC* represents the total number of generators, transformers, and shunt capacitors, respectively. Each control variable can be any number between its lower and upper limits. In practice, transformer Tap settings are discrete rather than continuous. However, in this study they are modeled as continuous variables in (p.u.) values, without explicitly considering the discrete voltage step magnitudes. This simplification is widely adopted in the OPF literature, as it facilitates comparison with previous works and ensures consistency across benchmark case studies.

### State variables (dependent)

The vector of state variables can be defined as follows^22^:

3 $$\:u=[{{Pg}_{1},Vl}_{1},\dots\:,{Vl}_{{N}_{D}};{Sl}_{1},\dots\:,{Sl}_{{N}_{l}};{Qg}_{1},\dots\:,{Qg}_{ng}]$$

Where,  *Pg*_1_ is the generator’s active power at slack bus, $\:{Vl}_{i}$ is the voltage of $\:{i}^{th}$ load bus and $\:{Sl}_{i}$ is the line loading of $\:{i}^{th}$ line. $\:{Qg}_{i}$ is the generator’s reactive power at the bus $\:i$, $\:{N}_{D}$ represents total number of load bus, $\:{N}_{l}$ represents total quantity of the lines.

### Constraints

As previously discussed, the OPF problem includes both equality and inequality constraints that must be met. These constraints are categorized and defined in the following sections.

#### Equality constraints

The power balance equations can be expressed as follows^33^:

4 $$\:{P}_{{g}_{i}}-{P}_{{d}_{i}}={V}_{i}\sum\:_{j=1}^{{N}_{b}}{V}_{j}[{G}_{ij}\mathrm{cos}\left({\theta\:}_{i}-{\theta\:}_{j}\right)+{B}_{ij}\mathrm{sin}\left({\theta\:}_{i}-{\theta\:}_{j}\right)]$$

5 $$\:{Q}_{{g}_{i}}+{Q}_{{C}_{i}}-{Q}_{{d}_{i}}={V}_{i}\sum\:_{j=1}^{{N}_{b}}{V}_{j}[{G}_{ij}\mathrm{sin}\left({\theta\:}_{i}-{\theta\:}_{j}\right)-{B}_{ij}\mathrm{cos}\left({\theta\:}_{i}-{\theta\:}_{j}\right)]$$

Where, $\:{N}_{b}$ is the total number of bus. $\:Pd$, $\:Qd$ are active and reactive load, respectively.$\:\:Pg$, $\:Qg$ are active and reactive power of generators connected to bus $\:i$, respectively. $\:{G}_{ij}$ is the conductance while $\:{B}_{ij}$ is the susceptance connecting the bus $\:i$ and $\:j$, respectively. The equality constraints are enforced through power flow calculations performed using the MATPOWER package.

#### Inequality constraints

In OPF, the inequality constraint represents the operational boundaries of the components within the electrical network, including restrictions on transmission lines and load buses, which are essential for maintaining the overall security and stability of the power system^33^.

*Generator constraints*:

6 $$\:\left\{\begin{array}{c}{Pg}_{i}^{max}\:\ge\:\:{Pg}_{i}\ge\:{Pg}_{i}^{min}\:\forall\:i\in\:ng\\\:{Qg}_{i}^{max}\:\ge\:\:{Qg}_{i}\ge\:{Qg}_{i}^{min}\:\forall\:i\in\:ng\end{array}\right.$$

*Transformer constraints*:

7 $$\:{T}_{i}^{max}\:\ge\:\:{T}_{i}\ge\:{T}_{i}^{min}\:\forall\:i\in\:nT$$

*Shunt compensator constraints*:

8 $$\:{Qc}_{i}^{max}\ge\:\:{Qc}_{i}\ge\:{Qc}_{i}^{min}\:\forall\:i\in\:nc$$

*Security constraints*:

9 $$\:\left\{\begin{array}{c}{V}_{i}^{max}\:\ge\:\:{V}_{i}\ge\:{V}_{i}^{min}\:\forall\:i\in\:{N}_{b}\\\:{Sl}_{i}^{max}\:\ge\:\left|{Sl}_{i}\right|\:\forall\:i\in\:{N}_{l}\end{array}\right.$$

The control variables associated with inequality constraints inherently impose their own limits. The optimization algorithm finds a good value for each of these variables and makes sure it is within the range that has been set. Section 3 goes into deep detail about the best ways to handle inequality constraints that have to do with state variables.

### Objective functions in OPF optimization

In this study, we used the test systems IEEE 30, 57, and 118-bus to perform a number of case studies on several objective functions to see how well different constraint handling methods work. The IEEE 30-bus system, smaller in scale, is employed for basic analysis, while the 57-bus system is used for moderate complexity studies. The IEEE 118-bus system, being larger and more detailed, simulates real-world and complex power systems, providing a more comprehensive analysis for advanced simulations and evaluations.

Table 1 shows a summary of the main parts, like generators, transformers, and shunt compensators, as well as other important parameters. Bus 1 is the slack bus in the 30-bus and 57-bus systems. It is also called the ($V\theta$) bus. In the 118-bus system, Bus 69 is the slack bus, on the other hand. The slack bus is very important for keeping the balance of active and reactive power in the system during power flow analysis by satisfying power. For clarity, the slack bus voltage magnitude (*V*) is set to 1 per unit (p.u.), and the voltage angle ($\theta$) is set to 0 degrees. The slack bus sets the voltage and angle for all the other bus, and the load flow study figures out what those values are. The next section describes the formulation of the study cases.

**Table 1:** Overview of test systems under study.

Item	IEEE 30-bus system	IEEE 57-bus system	IEEE 118-bus system
Number of buses	30	57	118
Bus details	^34^	^34^	^34^
Branches	41	80	186
Branch details	^34^	^34^	^34^
Number of generators	6 (Located at buses: 1 (slack), 2, 5, 8, 11, 13)	7 (Located at buses: 1 (slack), 2, 3, 6, 8, 9, 12)	54 (Located at buses: 1, 4, 6, 8, 10, 12, 15, 18, 19, 24, 25, 26, 27, 31, 32, 34, 36, 40, 42, 46, 49, 54, 55, 56, 59, 61, 62, 65, 66, 69 (slack), 70, 72, 73, 74, 76, 77, 80, 85, 87, 89, 90, 91, 92, 99, 100, 103, 104, 105, 107, 110, 111, 112, 113, 116)
Shunt VAR capacitors	9 (Located at buses: 10, 12, 15, 17, 20, 21, 23, 24, 29)	3 (Located at buses: 18, 25, 53)	14 (Located at buses: 5, 34, 37, 44, 45, 46, 48, 74, 79, 82, 83, 105, 107, 110)
Transformers	4 (Located at branches: 11, 12, 15, 36)	17 (Located at branches: 19, 20, 31, 35, 36, 37, 41, 46, 54, 58, 59, 65, 66, 71, 73, 76, 80)	9 (Located at branches: 8, 32, 36, 51, 93, 95, 102, 107, 127)
Number of control variables	24	33	130
Connected load	283.4 MW, 126.2 MVAr	1250.8 MW, 336.4 MVAr	4242 MW, 1439 MVAr
Voltage range for Load bus	24 buses with voltage: 0.95–1.05 p.u.	50 buses with voltage: 0.94–1.06 p.u.	64 buses with voltage: 0.94–1.06 p.u.

#### Fuel cost minimization

The primary objective function of OPF is widely investigated in the existing literature. It is commonly represented by a quadratic relationship between fuel costs (in $/h) and the generated power (in MW). Therefore, this objective function to be minimized can be expressed as follows^6^:

10 $$\:{C}_{0}\left({P}_{g}\right)={\sum\:}_{i=1}^{{N}_{g}}{a}_{i}+{b}_{i}{P}_{gi}+{c}_{i}{P}_{gi}^{2}$$

The cost coefficients, represented as *a*_i_, *b*_i_, and *c*_i_, correspond to the *i*^th^ generator’s cost for producing output power *P*_*gi*_. These coefficients for the generators in the IEEE 30-bus and IEEE 57-bus system is provided in Table 13 and Table 14 in the Appendix, respectively. While, cost coefficient of each generator for the 118 bus can be found in^34^.

#### Cost minimization considering multi-fuel sources

**Fig. 2:**
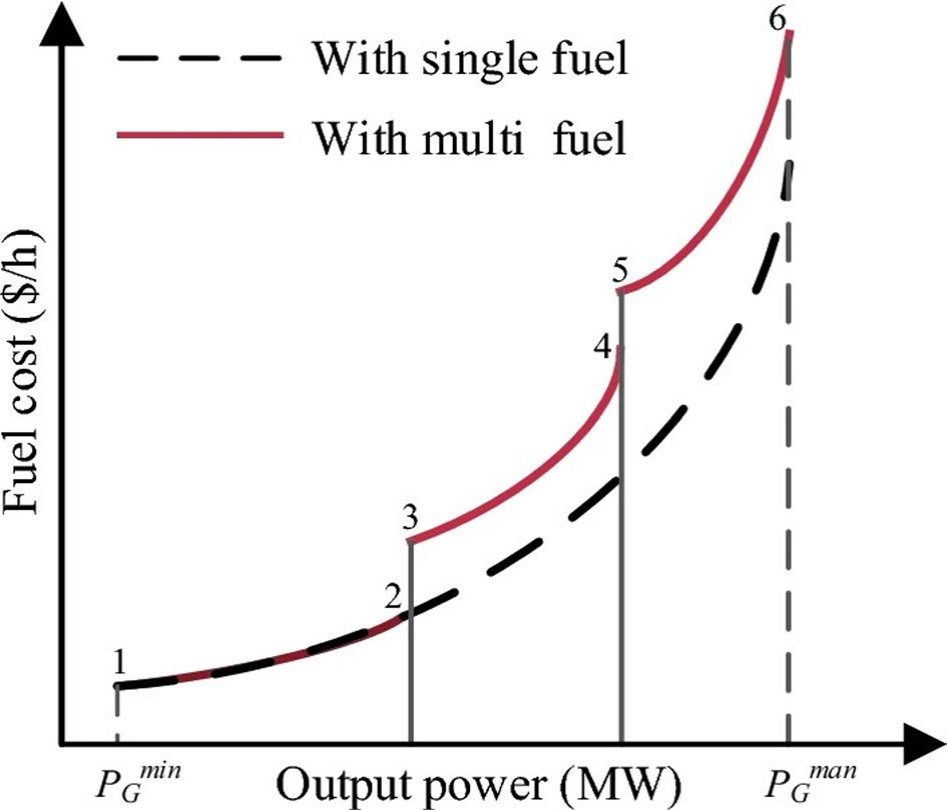
Generator cost profiles for single and multi fuel sources.

Thermal power plants use different types of fuel, such as coal, natural gas, and oil, to make each unit produce different amounts of power. In these cases, the fuel cost function is shown by a series of piecewise quadratic cost functions, as shown in Fig. 2. The exact shape of these functions depends on the type and number of fuels used.

The use of multiple fuels for the *i*^th^ generator incurs a cost, which can be expressed mathematically as^6^:

11 $$\:{C}_{0m}\left({P}_{g}\right)={\sum\:}_{i=1}^{{N}_{g}}{a}_{ij}+{b}_{ij}{P}_{gi}+{c}_{ij}{P}_{gi}^{2}$$

The power output for each fuel option *j* is constrained within the range: $\:{P}_{gi}^{min}\le\:{P}_{gi}\le\:{P}_{gi}^{max}$, where *j* represents the specific fuel type. In this system, the first two generating units are assumed to have the flexibility of using multiple fuel types. The associated coefficients and the power interval (in MW) are provided in Table 15 in the Appendix. For the rest of four generators, the cost structure remains the same with that of Case 1.

#### Voltage stability enhancement

Voltage stability has become an important problem in power systems, especially since there have been network failures caused by unstable voltages. When things are going well and when there are problems, the stability of a power system is based on how well it can keep the voltages at all buses within acceptable limits. When a system experiences voltage instability, it means that a disturbance, an increase in load demand, or any other change in system conditions causes the voltage to slowly and uncontrollably drop^35^. Voltage instability is more likely to happen in systems with long transmission lines and heavy loads. One of the main goals of power system management is to make voltage more stable. The Voltage Stability Index (VSI), defined as the maximum value of the *L*-index at each load bus, serves as an effective measure to assess the power system stability^36^. This index goes from 0 to 1, with 0 meaning the system is not under any load and 1 meaning the voltage drops. In an electrical network consisting of *N*_*L*_ load buses (PQ) and N_G_ generator buses (PV), the objective function can be expressed as follows^37^:

12 $$\:VSI=max\left|1-{\sum\:}_{i=1}^{{N}_{G}}{F}_{ji}\frac{{V}_{i}}{{V}_{j}}\right|$$

Where $$\:{F}_{ji}=-{\left[{Y}_{LL}\right]}^{-1}\left[{Y}_{LG}\right]$$ and j = 1,2,…, N_L_.

*N*_L_ is the number of load buses and *N*_G_ is the number of generator buses. Where, sub-matrices Y_LL_ and Y_LG_ are obtained from Y_BUS_ matrix after separating PQ and PV buses as represented in the following matrix^37^:

13 $$\:\left[\begin{array}{c}{I}_{L}\\\:{I}_{G}\end{array}\right]=\left[\begin{array}{cc}{Y}_{LL}&\:{Y}_{LG}\\\:{Y}_{GL}&\:{Y}_{GG}\end{array}\right]\left[\begin{array}{c}{V}_{L}\\\:{V}_{G}\end{array}\right]$$

#### Minimization of emission

The production of electricity from traditional energy sources results in the release of harmful gases into the atmosphere. As power generation increases, the emissions of sulfur oxides (SOx) and nitrogen oxides (NOx) per hour (t/h) also rise, as defined by the equation in Eq. (14). Therefore, reducing these emissions is established as an objective function of the OPF^6^:

14 $$\:E=\sum\:_{i=1}^{{N}_{G}}\left[\left({\alpha\:}_{i}+{\beta\:}_{i}{P}_{TGi}+{\gamma\:}_{i}{P}_{TGi}^{2}\right)\:\times\:\:0.01+{w}_{i}{e}^{{\mu\:}_{i}{P}_{TGi}}\right]$$

Where, the emission coefficients $\alpha_i$,$\beta_i$,$\gamma_i$,$\omega_i$, and $\mu_i$ are provided in Table 13 and Table 14 in the Appendix of the 30-bus and 57-bus, respectively.

#### Active power loss minimization

Active power loss in a power system is an essential problem because of the resistance present in the lines. The active power loss (MW) can be given as follows^37^:

15 $$\:{P}_{loss}=\sum\:_{k=1}^{{N}_{l}}{G}_{k}[{V}_{i}^{2}+{V}_{j}^{2}-2{V}_{i}{V}_{j}\mathrm{c}\mathrm{o}\mathrm{s}({\theta\:}_{i}-{\theta\:}_{j}\left)\right]$$

Where, $\:{\theta\:}_{i}-{\theta\:}_{j}$​ represents the voltage angle difference between bus *i* and *j*​. Additionally, *G*_*k*_ is the transfer conductance of the branch *k* that connects *i* and *j*​.

#### Fuel cost minimization considering valve-point loading impact

**Fig. 3:**
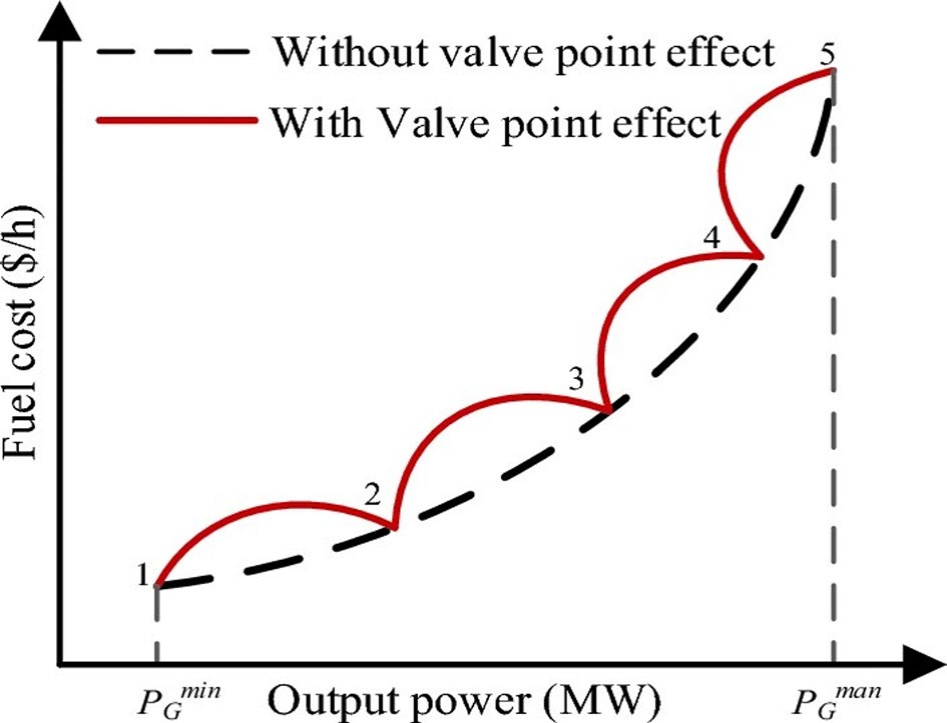
Generator cost profiles with and without the influence of valve-point loading.

The valve-point loading impact makes the generator’s cost function non-smooth, which makes the cost curve change. The steam flow to the turbine blades is regulated by multiple sets of nozzles. When valves open suddenly, the wire drawing effect makes fuel prices go up very quickly. Therefore, the cost curve will appear irregular, featuring fluctuations as depicted in Fig. 3. To show how much more this costs because of the valve-point effect, a sinusoidal function is used, and its modulus shows how much more it costs.

The objective function of fuel cost minimization considering valve-point loading impact is given as^6^:

16 $$\:C\left({P}_{g}\right)={\sum\:}_{i=1}^{{N}_{g}}{a}_{i}+{b}_{i}{P}_{gi}+{c}_{i}{P}_{gi}^{2}+\left|{d}_{i}\times\:\mathrm{sin}({e}_{i}\times\:({P}_{gi}^{min}-{P}_{gi}\left)\right)\right|$$

Where, the coefficients $d_i$, $e_i$signify the influence of the valve loading and they can be found in Table 13 in the Appendix.

#### Minimization of cumulative voltage deviation

Cumulative voltage deviation (CVD) is considered a key indicator of the quality of voltage within a power network. It plays a critical role in ensuring system security. This metric is defined as the total sum of the differences between the actual voltages at all load buses in the network and the reference value of unity. It can be defined as^37^:

17 $$\:CVD=\sum\:_{i=1}^{{N}_{L}}|{V}_{i}-{V}_{i}^{ref}|$$

Where, $\:{V}_{i}$ represents the bus voltage at the $\:{i}^{th}$ load bus, $\:{V}_{i}^{ref}$ is the reference value which is equal to 1 p.u.

## Integration of constraint handling (CH) methods into the QPSODM algorithm

### Particle swarm optimization (PSO) description

Particle Swarm Optimization (PSO) is an evolutionary computation method inspired by the collective behaviour of social organisms, as introduced by Kennedy and Eberhart^38^. This technique relies on how individual agents make decisions based on two key sources of information. The first is personal experience, each agent evaluates the choices it has previously made, identifying which option has yielded the most favourable outcome and how beneficial it was. The second source of information comes from the experiences of neighbouring agents. That is, each agent observes and learns from the performance of others, recognizing which choices have led to optimal results within the swarm and how effective those solutions have been. In a PSO framework, agents refine their decisions by considering both their own historical performance and the insights gained from their peers. The process begins with a population of randomly generated candidate solutions, each represented as a particle within the problem space. Each particle is assigned an initial velocity and moves through the search space, continuously updating its position. These agents retain memory, allowing them to track their personal best-known position (*P*_*best*_) along with its associated fitness value. Among all particles in the swarm, the one that has achieved the highest fitness value is designated as the global best (*G*_*best*_).

Mathematically, the swarm operates in an n-dimensional space, where the position of the *i*^*th*^ particle is denoted as $\:{X}_{i}=({x}_{i1},{x}_{i2},\dots\:,{x}_{in})$. The best position previously reached by this particle, which corresponds to its highest fitness value, is expressed as $\:{P}_{i1}=({p}_{i1},{p}_{i2},\dots\:,{p}_{in})$. The overall best position attained by any particle in the swarm is represented as $\:{P}_{g}=({p}_{g1},{p}_{g2},\dots\:,{p}_{gn})$. Additionally, each particle’s velocity, which determines the rate at which its position changes, is given by $\:{V}_{i}=({v}_{i1},{v}_{i2},\dots\:,{v}_{in})$. The movement and adaptation of particles are governed by specific mathematical equations that dynamically adjust their velocities and positions based on both individual and collective knowledge, driving the swarm toward optimal solutions. The particles are controlled according to the following equations^33^:

18 $$\:{v}_{i}^{t+1}={w}^{t}.{v}_{i}^{t}+{c}_{1}.{r}_{1}.\left({{P}_{i}^{t}-x}_{i}^{t}\right)+{c}_{2}.{r}_{2}.\left({{P}_{g}^{t}-x}_{i}^{t}\right)$$

19 $$\:{x}_{i}^{t+1}={x}_{i}^{t}+{v}_{i}^{t+1}$$

Here, i ranges from 1 to N, where N represents the total number of individuals in the population. The parameter w represents the inertia weight, which is determined by the following expression^33^:

20 $$\:{w}^{t}={w}_{max}-\left(\frac{{w}_{max}-{w}_{min}}{{t}_{max}}\right)t$$

The parameters governing the velocity update include the initial weight $\:{w}_{max}$, the final weight $\:{w}_{min}$​, the current iteration index $\:t$, and the maximum number of iterations $\:{t}_{max}$​.

Two positive constants, commonly denoted as $\:{c}_{1}$​ and $\:{c}_{2}$​, represent the cognitive and social learning factors, respectively. As reported by^39^, typical values assigned to these parameters are $\:{w}_{max}=0.9$, $\:{w}_{min}=0.4$, $\:{c}_{1}={c}_{2}=2$, and these values are generally chosen regardless of the specific optimization problem. The terms $\:{r}_{1}$​ and $\:{r}_{2}$​ refer to random variables drawn from a uniform distribution over the interval (0, 1).

**Fig. 4:**
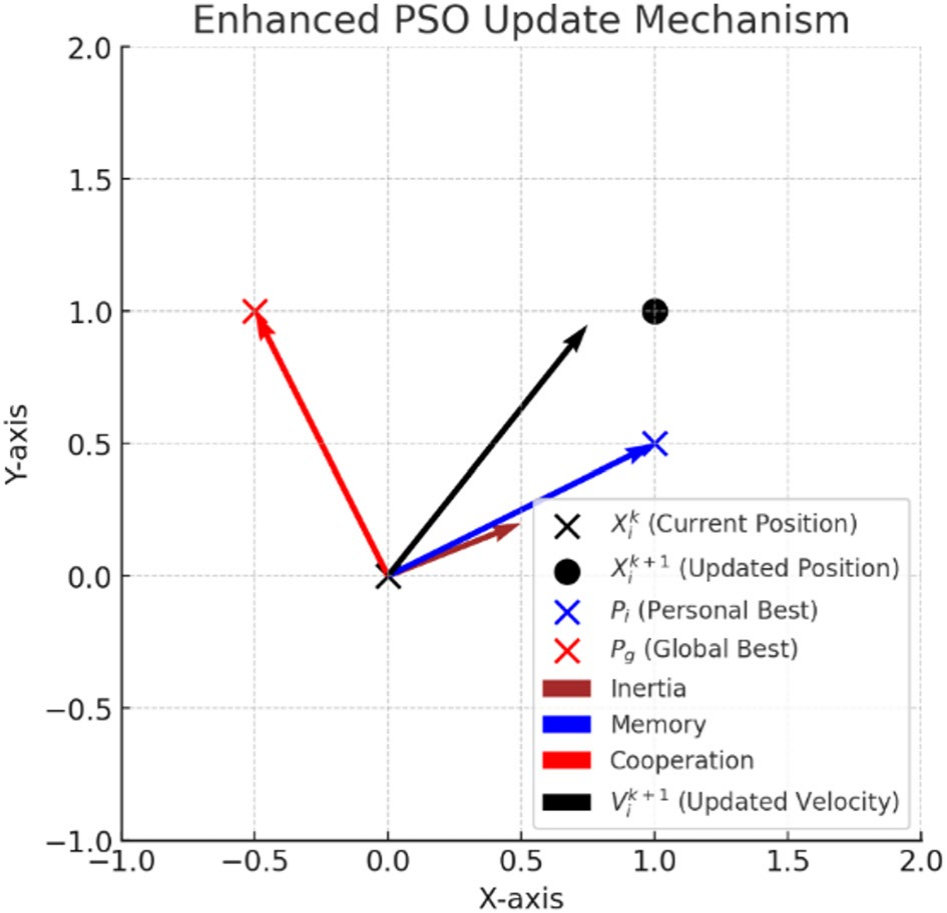
Enhanced PSO velocity and position update mechanism.

Equation (18) is employed to calculate the updated velocity $\:{v}_{i}^{t+1}$​ for particle $\:i$ at each iteration, whereas Eq. (19) determines the updated position $\:{x}_{i}^{t+1}$​ by adding the newly computed velocity to the current position $\:{x}_{i}^{t}$​. Figure 4 illustrates how velocity and position are updated in a two-dimensional search space.

### Quantum behaved particle swarm optimization (QPSO) description

One of the principal limitations of the PSO algorithm is its inability to guarantee global convergence. To alleviate this concern, the Quantum-behaved Particle Swarm Optimization (QPSO) framework was introduced, with early developments presented in studies by^40^. Theoretically, convergence in PSO can be achieved when each particle moves toward its local attractor, denoted by $\:{A}_{i}=({A}_{i1},{A}_{i2},\dots\:,{A}_{in})$., which is derived from the particle’s own best-known position and the global best. As introduced by^41^, the attractor can be given as^40^:

21 $$\:{A}_{i}^{t}=u\:.{P}_{i}^{t}+\left(1-r\right){P}_{g}^{t}$$

​

where $\:r\in\:\left(\mathrm{0,1}\right)$ is a random variable. This formulation defines a stochastic attractor that lies within the hyper-rectangle spanned by the individual best ($\:{P}_{i}$​) and global best ($\:{P}_{g}$​​) positions. Over time, as particles move closer to these attractors, their trajectories tend to merge. Consequently, the individual bests, the attractors, and the global best converge to a common point, thereby ensuring convergence of the entire swarm. In the Quantum-behaved Particle Swarm Optimization (QPSO) framework, each particle’s state is described by a *wave function* $\Psi$ rather than by explicit positional and velocity vectors, as is typical in classical PSO models. This quantum representation introduces a fundamentally different dynamic, where the exact position and velocity of a particle cannot be simultaneously determined. Instead, what can be known is the *probability distribution* of a particle’s location, determined by the squared magnitude of its wave function,$\:\:{\left|{\Psi}\right|}^{2}$. This probability density is influenced by the potential landscape in which the particle exists. At a given iteration *t*, if particle *i* is assumed to be moving within an N-dimensional space and is situated in a potential well cantered at its personal best position $\:{A}_{i}^{t}$​, its wave function at the next iteration can be expressed as^40^:

22 $${\Psi}\left({x}_{i}^{t+1}\right)=\frac{1}{\sqrt{{D}_{i}^{t}}}\mathrm{e}\mathrm{x}\mathrm{p}(-\frac{\left|{x}_{i}^{t+1}-{A}_{i}^{t}\right|}{{D}_{i}^{t}})$$

This leads to a double exponential probability density function^40^:

23 $$\:\mathrm{Q}\left({x}_{i}^{t+1}\right)={\left|{\Psi}\left({x}_{i}^{t+1}\right)\right|}^{2}=\frac{1}{{D}_{i}^{t}}\mathrm{e}\mathrm{x}\mathrm{p}(-2\frac{\left|{x}_{i}^{t+1}-{A}_{i}^{t}\right|}{{D}_{i}^{t}})$$

where $\:{D}_{i}^{t}$ is the standard deviation of the double exponential density function. From this density, a cumulative distribution function $\:\mathrm{F}\left({x}_{i}^{t+1}\right)$ can also be derived. To sample the next position of the particle, a Monte Carlo approach is applied^40^:

24 $$\:{{x}_{i}^{t+1}=A}_{i}^{t}\pm\:\frac{{D}_{i}^{t}}{2}\mathrm{ln}\left(\frac{1}{r}\right)$$

The scale parameter $\:{D}_{i}^{t}$​, which manages the spread of the exponential distribution, can be written as^40^:

25 $$\:{D}_{i}^{t}=2\:.\:\beta\:\:.\:\left|{M}_{best}^{t}-{x}_{i}^{t}\right|$$

Here, $\:{M}_{best}^{t}$ is the mean best position, calculated as the average of the best-known positions $\:{P}_{best}$ of all particles in the swarm^40^:

26 $$\:{M}_{best}^{t}=(1/Ps){\sum\:}_{i=1}^{Ps}{P}_{i}^{t}$$

Where, *N* represents the total number of particles. Therefore, the position update formula in QPSO becomes^40^:

27 $$\:\left\{\begin{array}{c}{{x}_{i}^{t+1}=A}_{i}^{t}+\beta\:\:\left({M}_{best}^{t}-{x}_{i}^{t}\right)\mathrm{ln}(\frac{1}{r})\:\:\:\:\:if\:\:\:z>0.5\\\:{{x}_{i}^{t+1}=A}_{i}^{t}-\beta\:\:\left({M}_{best}^{t}-{x}_{i}^{t}\right)\mathrm{ln}(\frac{1}{r})\:\:\:\:\:if\:\:\:z\le\:0.5\end{array}\right.$$

Where $\:z$ is a random number uniformly distributed in the range [0, 1]. The sole parameter in QPSO is the contraction expansion factor $\:\beta\:$, which adjusts the behaviour of the convergence of the algorithm and is typically fixed to a value not more than $\:1.7$ for performance and stability^40^. Due to its effectiveness and simplicity of implementation, QPSO has been successfully applied across a series of standard optimization problems with notable results.

### Quantum behaved PSO with differential mutation (QPSODM)

Differential Evolution (DE), a method based on population by^42^, is a collaborative and competitive optimization technique applied to a candidate solution. It has proven to be useful for stubbornly complex problems, particularly optimization problems with erratic non-differentiable objective functions. The algorithm continuously improves the solution set, or population, using some straightforward arithmetic and evolutionary operations such as mutation, crossover, and selection. DE refines the population through mutations from random initial states towards optimal or near-optimal solutions starts from a random population. According to^43^, the implementation of the mutation strategy is crucial for the robustness of diversity as well as convergence performance:

28 $$\:{x}_{i}^{t+1}={x}_{j}^{t}+\left(1-\alpha\:\right)\times\:\left({x}_{k}^{t}-{x}_{l}^{t}\right)+\alpha\:\times\:\left({{P}_{g}^{t}-x}_{j}^{t}\right)$$

Where *j*,* k*,* l* are random integers uniformly chosen from [1 : N] and $\:i\:\ne\:\:j\:\ne\:\:k\:\ne\:\:l$. While $\:\alpha\:$ can be defined as follow^43^:

29 $$\:\alpha\:=\frac{{t}_{act}}{{t}_{max}}$$

Where $\:{t}_{act}$ is the actual iteration and $\:{t}_{max}$ is the maximum iteration.

The flowchart illustrating the PSO, QPSO, and QPSODM algorithms is presented in Fig. 4.

### Constraint handling (CH) methods

By using the knowledge from infeasible solutions, a suitable metaheuristic algorithm in conjunction with a suitable constraint handling strategy can direct the search process towards globally feasible solutions. The static penalty approach, which penalizes the fitness of impractical solutions based on constraint breaches, is one of the most used techniques for addressing constraints. Although this method is easy to use and straightforward, its effectiveness largely depends on the penalty factor, which often requires manual fine-tuning through a process of trial and error. Below are some alternate CH strategies that are integrated with our algorithm to remove the necessity for such fine-tuning. A constrained optimization problem involving the optimization of *D* parameters is generally expressed as follows^22^:

**Objective**:

$$\:Minimize:f\left(x\right)\:where\:x=\left({x}_{1},{x}_{2},\dots\:{,x}_{n}\right)$$

30 $$\mathrm{S}\mathrm{u}\mathrm{b}\mathrm{j}\mathrm{e}\mathrm{c}\mathrm{t}\:\mathrm{t}\mathrm{o}:\left\{\begin{array}{c}{g}_{i}\left(x\right)\le\:0,\:\:i=[1:n]\\\:{h}_{j}\left(x\right)=0,\:\:j=[n+1:m]\end{array}\right.$$

Where, $\:f\left(x\right)\:$ represents the objective function to be minimized, while the constraints guarantee that the solution keeps within feasible bounds. The inequality constraints $\:{g}_{i}\left(x\right)$ and equality constraints $\:{h}_{j}\left(x\right)$ define the allowable solution in the solution space. Where $\:n$​ represents the total number of inequality constraint, while ($\:m-n$) represents the count of equality constraint. It defines the overall set of restrictions that must be satisfied for a feasible solution. The equality constraints are converted into inequality constraints. After normalizing all constraints, the overall constraint representation is expressed as^22^:

31 $$\:{G}_{i}\left(x\right)=\left\{\begin{array}{c}max[{g}_{i}\left(x\right),0],\:\:\:\:\:\:\:\:\:\:\:\:\:\:\:\:\:i=[1:n]\\\:max[{h}_{i}\left(x\right)-\theta\:,0],\:\:i=[n+1:m]\end{array}\right.$$

The parameter $\:\theta\:$ represents the tolerance level of the equality constraint. The objective functions *f(x)* is designed to be optimized, ensuring that optimal resulting solutions adheres to inequality constraint $\:{G}_{i}\left(x\right)$. In cases where a solution does not meet feasibility conditions, the total constraint violation is evaluated as the weighted average of all constraint deviations, expressed as^22^:

32 $$\:\varDelta\:\left(x\right)=\frac{{\sum\:}_{i=1}^{m}{w}_{i}\left({G}_{i}\left(x\right)\right)}{{\sum\:}_{i=1}^{m}{w}_{i}}$$

where, $\:{w}_{i}=1/{G}_{i}^{max}$. In this context, $\:{w}_{i}$ represents a weighting factor, while $\:{G}_{i}^{max}$ denotes the upper limit for constraint violation associated with $\:{G}_{i}\left(x\right)$. Within the algorithm, the weight $\:{w}_{i}$ is defined as $\:{1/G}_{i}^{max}$​, allowing it to dynamically adjust throughout the optimization process. This adaptive approach ensures that each constraint contributes proportionally to the problem formulation, despite variations in their numerical magnitudes. Additionally, this study provides a concise overview of three CH methods usually used in constrained optimizing problems of the power systems.

#### **Epsilon () constraint (ECO)**

In the *ε-*constraint handling (ECO) approach^44^, the overall constraint violation is regulated through the *ε-*parameter. This method proves particularly effective when dealing with optimization problems that involve active constraints. By adjusting the *ε-*parameter, the search process can be guided efficiently toward feasible solutions^44^. The value of ε is iteratively updated until the iteration counter (*t*) reaches a predefined control iteration bound, *C*_*t*_. Once this threshold is exceeded, ε is set to zero, ensuring that the obtained solutions fully comply with all constraints. The updating strategy for the *ε-*parameter follows the equations outlined below^44^:

33 $$\:\epsilon\:\left(0\right)=\varDelta\:\left({x}_{\rho\:}\right)$$

34 $$\:\epsilon\:\left(t\right)=\left\{\begin{array}{c}\epsilon\:\left(0\right)\times\:{(1-\frac{t}{{C}_{t}})}^{cp},\:\:0<t<{C}_{t}\\\:0,\:\:t\ge\:{C}_{t}\end{array}\right.$$

The top-ranked individual, denoted as $\:{x}_{\rho\:}$, corresponds to the $\:{\rho\:}^{th}$ percentile of the population, where $\:\rho\:$ is determined as 5% of the total population size P_S_. The parameter *t* represents the current generation count, while *C*_*t*_ specifies the number of iterations for which the *ε-*parameter is actively controlled before being set to zero. Recommended parameter values include cp. ranging from 2 to 10 and *C*_*t*_ varying from 10% to 80% of the maximum allowed iterations ($\:{t}^{max}$). In ECO, individual selection is made by comparing one solution to another, using the same rules as in the SFS method. The key difference in ECO is that an optimal solution is considered feasible only if its total constraint violating less than the current $\:\varepsilon\:\left(t\right)$, rather than requiring a violation of zero. Initially, $\:\varepsilon\:\left(t\right)$ is set to a small value, which is gradually reduced to zero over time.

#### Superiority of feasible solutions (SFS)

The SFS approach^45^ establishes a comparison framework between two candidate solutions. A solution *x*_*i*​_ is considered superior to another solution *x*_*j*_​ under the following conditions:

- *x*_*i*​_​ is feasible, whereas *x*_*j*_​ is not.
- Both solutions are feasible, but *x*_*i*​_​​ achieves a lower objective function value in the context of a minimization problem.
- If neither solution is feasible, the one with the lesser total constraint violation is considered superior and can be defined as $\:\varDelta\:\left({x}_{i}\right)<\varDelta\:\left({x}_{j}\right)$ according to Eq. (32).

This method always gives priority to workable ideas over impractical ones. Two viable solutions are compared purely on the basis of their objective function values. On the other hand, the degree of constraint violations is used to evaluate both options if they are not practicable. By reducing their constraints, this approach pushes impractical options closer to viability. In the meantime, evaluating workable options according to their objective values improves the quality of the solution as a whole.

#### Stochastic ranking (SRA)

In a constrained optimization problem, achieving a balance between minimizing the objective function and ensuring whole constraint satisfaction is essential for converging to a globally optimal feasible solution. To address this challenge, a stochastic approach known as stochastic ranking (SRA) has been introduced in^46^. This method uses a probability factor ($\:Pf$) to decide whether the ranking of a solution will be based on its objective function value or its degree of constraint violation. The SRA process can be outlined as follows:

- *If there are no violations of the constraints*,* or if the random value is less than the probability factor*,
- *Rank the solutions solely based on their objective values.*
- *Otherwise*,
- *Rank the solutions according to the degree of constraint violations.*

Either the objective function values or the degree of constraint violations, which are chosen at random, are used in the SRA approach to compare individuals. This strategy finally directs the search towards a feasible region that contains the global optima by allowing impractical solutions with higher objective values to endure throughout the process of evolution. It has been demonstrated that SRA’s CH method works very well in optimisation problems with disconnected viable zones. Furthermore, rather from maintaining the probability factor ($\:Pf$) constant, research indicates that SRA’s efficacy increases when it is gradually reduced from 0.475 in the first iteration to 0.025 in the last iteration^46^. In particular, the probability for any given iteration (t > 1) is determined by modifying the value of the preceding iteration in the manner described below^46^:

35 $$\:Pf\:\left(t\right)=Pf\:\left(\mathrm{t}-1\right)-\frac{Pf\:\left(1\right)-Pf\:\left({t}^{max}\right)}{{t}^{max}\:-\:1}$$

where $\:Pf\:\left(1\right)=0.025$ for the first iteration (*t = 1*) and $\:Pf\:\left({t}^{max}\right)=0.025$ for the final iteration ($\:t={t}^{max}$).

### CH techniques integrated with QPSODM

This section outlines the incorporation of the optimization algorithm QPSODM, applied separately with each constraint handling technique. No explicit constraints management method is included in the basic form of the PSO algorithm^47^. Yet, some CH techniques can be borrowed from the general approaches for EAs, while others are designed considering specific details of PSO that can be exploited to improve its performance. Although there are attempts in the literature to classify these techniques, it is rarely straightforward to assign them to a particular class, especially in terms of the PSO paradigm. The selection procedure in both QPSODM (and PSO) is unconstrained by default. However, when QPSODM is paired with the CH method, the process of selecting individuals for the subsequent iteration adheres to the specific guidelines of the chosen CH method.

**Fig. 5:**
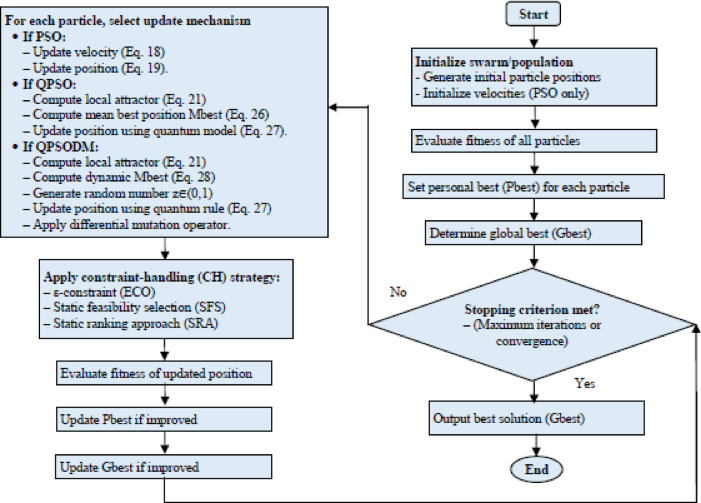
Flowchart for the implementation of QPSODM-XX (XX: ECO/SFS/SRA).

A detailed explanation of the combined QPSODM-XX (where XX represents ECO/SFS/SRA) algorithm is provided in Table 2, accompanied by a flowchart depicted in Fig. 5. While Table 2 outlines the process in full, the flowchart offers a high-level summary of the key steps. It is important to highlight that STEP 4 (the selection steps) in the (algorithm loop), as shown in Table 2, varies depending on the CH method applied, as well as any parameters unique to the selected CH technique.

**Table 2:** Pseudocode of the integration of the 3-CH methods with QPSODM algorithm.

QPSODM-XX (XX: ECO/SFS/SRA)	
INPUT SETTING ASSESMENNT ALGORITHM LOOP: STEP 1 STEP 2 STEP 3 STEP 4 STEP 5	- Problem dimension (d). - Population size, Ps. - Termination condition, maximum number of iterations, $\:{t}^{max}$. - Upper and lower bounds of decision (control) variables, vector form (X_min_ and X_max_). Where, X_min_ = [$\:{X}_{min}^{1}$, …, $\:{X}_{min}^{d}$] and X=[$\:{X}_{max}^{1}$, …, $\:{X}_{max}^{d}$]. - Set the iteration counter as t = 1. - Set QPSODM parameters: $\:\beta\:=1.2$. - Generate a population of Ps individuals, ensuring they are uniformly distributed within the defined bounds [X_in_, X_max_]. - Set the initial parameters of CH technique: Set $\:\epsilon\:=0$ for ECO, Eq. (33). Set Pf(1) = 0.475 and Pf($\:{t}^{max}$) = 0.025 for SRA, Eq. (35). The SFS technique does not require any predefined parameters. - Set Pbest and Gbest - Assess the objective function Eq. (30), constraint function Eq. (31) and constraint violation Eq. (32), for every particle *x*_k_ where $\:k\in\:$ [1, Ps]. - Increment the function evaluation count by Ps. - Adjust the parameters of the CH techniques, ($\:\epsilon\:$ parameter for ECO and *P*_*f*_ for SRA). - Calculate the local attractor according to Eq. (21). - Calculate *M*_*best*_ according to Eq. (26). - Generate z as a random number in a range [0, 1]. - If z < 0.5 - Update particle position according to Eq. (27). - Else - Update particle position according to Eq. (28). - Calculate the objective function Eq. (30), constraint function Eq. (31) and constraint violation Eq. (32), for every particle _*k*_ where $\:k\in\:$ [1, Ps]. - Increment the function evaluation count by Ps according to principles of the CH technique. ***QPSODM-ECO***: A particle is supposed as superior to its corresponding member in the existing population if it results in a lower degree of constraint violation. In addition, the particle is also supposed better if it satisfies the feasibility threshold, either by attaining zero constraint violation or by remaining under a pre-defined tolerance level ε, as defined in Eq. (34). In such cases, when constraint violation is insignificant, the selection further favoritisms the solution with a lower objective function value, in line with the minimization problem. ***QPSODM-SFS***: A particle is supposed superior if it exhibits a lower constraint violation or fully satisfies all constraints while achieving a smaller fitness value compared to its corresponding member in the previous population. ***QPSODM-SRA***: A particle is supposed superior if it achieves zero constraint violation in a given iteration while also yielding a lower fitness value compared to the corresponding member of the previous population. However, a particle with a lower fitness but a greater constraint violation might still be chosen for the next iteration, provided that a randomly number (*rand*) is smaller than the current iteration’s SRA probability factor (*P*_*f*_). Conversely, if *rand* exceeds *P*_*f*_, the particle exhibiting lower constraint violation takes precedence and replaces the existing population member. - In all other scenarios where the particle performs poorly, the existing population member remains unchanged to preserve better solutions. - If the condition is met, *t > t*_*max*_, STOP. - Otherwise, increment the iteration counter ($\:t=t+1$) and return to the first step of the algorithm loop.

### Parameter settings and justification

The population size was selected based on commonly adopted practices in swarm-based optimization for OPF problems and preliminary feasibility-oriented trials. Initial experiments indicated that excessively large populations may increase the frequency of constraint violations, particularly in highly constrained OPF formulations, without improving objective values. Therefore, moderate population sizes were adopted to ensure stable convergence and high feasibility rates, as later confirmed by the results reported in Section 4. The maximum number of iterations was defined to provide a conservative upper bound that guarantees convergence stability and feasibility across all tested systems and constraint-handling strategies. Preliminary convergence observations showed that most algorithms reached stable solutions well before the imposed iteration limit, indicating that this value does not artificially inflate computational effort but rather ensures robustness under different operating conditions. Using the same iteration limit for all compared methods also ensures a fair and unbiased performance comparison. To assess the robustness of the proposed framework with respect to parameter selection, a sensitivity analysis was conducted by varying the population size and the maximum number of iterations within reasonable ranges commonly reported in the literature. The results showed that moderate parameter variations did not alter the relative performance ranking of the compared methods, and no significant improvements were observed beyond the selected values. These observations confirm that the reported results are stable and not critically dependent on fine parameter tuning.

## Case studies and simulation results

This section presents the outcomes of case studies and simulation analyses for the QPSODM-SFS, QPSODM-ECO, and QPSODM-SRA algorithms. The results are organized in tabular form, accompanied by a comprehensive system-by-system explanation and comparative evaluation. The proposed algorithms were realized employing MATLAB, with simulation performed using a system equipped with an Intel Core i7 processor (2.7 GHz) and 12GB RAM. User-defined input parameters for the algorithms are detailed in Table 3, while Table 4 provides an overview of the objectives associated with different case studies, highlighting the optimization parameters for each test system. The population sizes listed in Table 3 were determined through multiple preliminary trial runs. It was observed that increasing the population size beyond the reported values did not improve solution quality and, in several cases, led to reduced feasibility rates due to increased constraint violations. These observations confirm that the selected population sizes provide an effective balance between exploration capability and feasibility preservation.

**Table 3:** Key parameters for the QPSODM-XX (XX: ECO/SFS/SRA) algorithms.

Parameters	IEEE 30 bus	IEEE 57 bus	IEEE 118 bus
Problem dimension (d)	24	33	130
Population size (Ps)	50	50	100
Maximum number of iterations (t^max^)	500	600	2000
Decision (control) variables	Refer to results, (X_min_ … X_max_)		
Constant (cp), for ECO technique only	5	5	5

Both IEEE 30-bus and IEEE 57-bus test systems require almost 500 iterations for each algorithm run. Given the higher number of control variables in the IEEE 118-bus system, a stopping criterion of 2000 iterations was carried out. To ensure statistical reliability and assess the performance of the 3-CH methods, each case study was executed independently 20 runs.

**Table 4:** Overview of the different case studies in OPF.

Parameter	Case 1	Case 2	Case 3	Case 4	Case 5	Case 6	Case 7	Case 8	Case 9	Case 10	Case 11
Test System	**IEEE 30 bus**						**IEEE 57 bus**			**IEEE 118 bus**	
Fundamental Fuel Cost	☑					☑	☑			☑	
Multiple-Fuel Cost		☑									
Valve-Loading Effect						☑					
Carbon Emission				☑							
Real Power Loss					☑				☑		☑
Cumulative Voltage Deviation								☑			
Voltage Stability Index			☑								

### Statistical comparison of QPSODM-ECO, QPSODM-SFS and QPSODM-SRA algorithms

Table 5 presents a statistical summary of 20 independent runs conducted for each of the 11 case studies, employing various CH methods considering QPSODM as the foundational optimizer. This table provides key performance indicators, including worst, mean, best and standard deviation (SD) values across all cases. Notably, no single algorithm consistently achieves the best fitness or mean performance across all instances. To further investigate the comparative performance of these algorithms, we employ the Wilcoxon signed-rank test^48^, a non-parametric statistical test used to assess significant differences between two paired datasets. The procedure for comparing the performance of two algorithms, denoted as ‘Al1’ and ‘Al2,’ within a specific case study is as follows:

Collect the fitness values obtained over 20 runs for both algorithms under consideration.

1. Determine $\:{R}^{+}$, representing the total of rank scores corresponding to instances where algorithm Al1 performs better than algorithm Al2.
2. Similarly, calculate $\:{R}^{-}$, which denotes the cumulative rank scores for cases where algorithm Al2 achieves superior performance compared to algorithm Al1.
3. Determine the *p*-value, which indicates the statistical significance of the results. A smaller *p*-value provides stronger evidence against the null hypothesis (*H*_*0*_​), suggesting a meaningful performance difference between the two algorithms.

**Table 5:** An analytical overview of case studies employing the 3-CH methods.

CH Case	QPSODM-ECO				QPSODM-SFS				QPSODM-SRA			
	Worst	Mean	Best	SD	Worst	Mean	Best	SD	Worst	Mean	Best	SD
Case 1	800.5103	**800.5084**	**800.4129**	0.00023	800.5312	800.5121	800.4130	0.00053	800.5458	800.5221	800.5121	0.00642
Case 2	646.6071	646.5993	**646.1331**	0.01253	647.6247	**646.5849**	646.5882	0.00732	647.6320	646.8171	646.7373	0.05854
Case 3	0.13782	0.13367	0.13633	0.00004	0.13672	**0.13646**	**0.13627**	0.00007	0.13901	0.13828	0.13742	0.00101
Case 4	0.20482	**0.20481**	**0.20481**	0.00001	0.20482	**0.20481**	**0.20481**	0.00001	0.20482	**0.20481**	**0.20481**	0.00001
Case 5	3.08842	**3.08573**	**3.08321**	0.00013	3.08843	**3.08573**	**3.08321**	0.00014	3.12187	3.13472	3.10091	0.00065
Case 6	832.1007	832.0989	832.0709	0.00198	832.0915	**832.0834**	**832.0691**	0.00353	834.5238	833.4215	832.4142	0.33845
Case 7	41667.22	41666.06	41666.12	0.29646	41666.23	**41665.89**	**41665.78**	0.21154	41678.34	41673.49	41670.87	2.34541
Case 8	0.5934	**0.5884**	**0.5835**	0.00184	0.5962	0.5887	0.5849	0.00372	0.6378	0.6198	0.6012	0.00736
Case 9	9.9018	**9.8812**	**9.8706**	0.00732	9.8225	9.8023	9.7720	0.03854	10.2519	10.0834	9.9718	0.07520
Case 10	128743.8	**127996.7**	**127895.8**	7.04181	130547.8	130048.1	129658.6	31.1519	135941.2	135527.8	135350.3	68.3425
Case 11	17.1202	**16.6808**	**16.46353**	0.19801	17.8409	17.5751	17.06636	0.23592	20.45301	19.64229	18.38184	0.53324

The results of the Wilcoxon signed-rank test are summarized in Table 6. The *H₀* label represents the validity of the null hypothesis. If *H₀* is marked as “0” at a significance level of α = 5%, it suggests that the performance of the two techniques being compared is statistically equivalent for that case study.

In addition to hypothesis testing, effect size was quantified using the rank-biserial correlation *r*_*rb*_​, which complements the Wilcoxon test by assessing the practical significance of observed performance differences beyond statistical significance alone.

The comparison between QPSODM-SFS and QPSODM-ECO shows that both approaches exhibit statistically similar performance in most cases, which is further supported by small or negligible values of *r*_*rb*_​​, indicating limited practical differences. Exceptions are observed in Case 1, Case 8, and Case 10, where statistically significant differences are accompanied by large effect sizes, confirming the practical superiority of QPSODM-ECO in these scenarios. In contrast, QPSODM-SRA is consistently outperformed by both QPSODM-SFS and QPSODM-ECO across almost all test cases. This dominance is reflected not only by the rejection of the null hypothesis but also by consistently large values of *r*_*rb*_​​, highlighting substantial and practically meaningful performance gaps. The only exception is Case 4, where all three algorithms yield statistically equivalent results with negligible effect sizes, indicating comparable behavior. A notable observation in the comparison between QPSODM-SFS and QPSODM-ECO is that, despite their statistical similarity in several cases, neither algorithm uniformly achieves the best minimum and mean values across all scenarios. However, for larger-scale systems involving a higher number of decision variables, QPSODM-ECO tends to exhibit medium to large effect sizes in its favor, suggesting improved scalability and more robust optimization performance under increased problem complexity.

**Table 6:** Results of Wilcoxon signed-rank test.

Case	QPSODM-ECO vs. QPSODM-SFS						QPSODM-ECO vs. QPSODM-SRA						QPSODM –SFS vs. QPSODM -SRA					
	*R* ^+^	*R* ^-^	*p*-value	H₀	*r*_rb_	Effect	*R* ^+^	*R* ^-^	*p*-value	H₀	*r*_rb_	Effect	*R* ^+^	*R* ^-^	*p*-value	H₀	*r*_rb_	Effect
1	197	13	0.0006	1	0.88	Large	210	0	8.8 × 10^−5^	1	1.00	Large	210	0	8.8 × 10^−5^	1	1.00	Large
2	119	91	0.6012	0	0.13	Small	210	0	8.8 × 10^−5^	1	1.00	Large	210	0	8.8 × 10^−5^	1	1.00	Large
3	91	119	0.6012	0	−0.13	Small	210	0	8.8 × 10^−5^	1	1.00	Large	210	0	8.8 × 10^−5^	1	1.00	Large
4	0	0	1.0000	0	0.00	Negligible	0	0	1.0000	0	0.00	Negligible	0	0	1.0000	0	0.00	Negligible
5	107	103	0.9405	0	0.02	Negligible	210	0	8.8 × 10^−5^	1	1.00	Large	210	0	8.8 × 10^−5^	1	1.00	Large
6	88	122	0.5257	0	−0.16	Small	210	0	8.8 × 10^−5^	1	1.00	Large	210	0	8.8 × 10^−5^	1	1.00	Large
7	90.5	119.5	0.5883	0	−0.14	Small	210	0	8.8 × 10^−5^	1	1.00	Large	210	0	8.8 × 10^−5^	1	1.00	Large
8	167.5	42.5	0.0196	1	0.60	Large	210	0	8.8 × 10^−5^	1	1.00	Large	210	0	8.8 × 10^−5^	1	1.00	Large
9	157	53	0.0522	0	0.50	Medium	210	0	8.8 × 10^−5^	1	1.00	Large	210	0	8.8 × 10^−5^	1	1.00	Large
10	198	12	0.0005	1	0.89	Large	210	0	8.8 × 10^−5^	1	1.00	Large	210	0	8.8 × 10^−5^	1	1.00	Large
11	140.5	69.5	0.1851	0	0.34	Medium	210	0	8.8 × 10^−5^	1	1.00	Large	210	0	8.8 × 10^−5^	1	1.00	Large

Several factors influence the effectiveness of constraint-handling (CH) techniques. This involves factors such as the characteristics of the objective function, the complexity of the constraint, and the ratio of the feasible to infeasible region within the solutions space, and internal mechanisms of the optimization method itself. The QPSODM-SFS approach, for instance, may struggle to maintain population diversity and is prone to stagnation^49^, particularly when the feasible region constitutes only a small fraction of the overall search space, or when the initial population contains very few feasible solutions. In such cases, the scarcity of feasible solutions in the early stages of the searching process can stop QPSODM-SFS from achieving optimal values during trial runs. Although QPSODM-ECO and QPSODM-SFS employ the same selection principles, QPSODM-ECO includes a feature that permits the selection of infeasible solutions with some level of constraint violation during the early stages of the search process, based on the epsilon ($\epsilon$) value. This approach enhances the exploration of the solution space, particularly near the boundary between feasible and infeasible regions, allowing for a more thorough search and potentially better outcomes. On the other hand, QPSODM-SRA is generally recommended for problems characterized by disconnected feasible regions^50^, as it utilizes a probabilistic operator capable of selecting infeasible solutions with higher constraint violations if they exhibit promising fitness values at any stage of the evolutionary process. However, the findings indicate that QPSODM-SRA is not as effective as QPSODM-SFS and QPSODM-ECO when applied to the OPF problem, particularly in large-scale electrical networks. The statistical analysis suggests that selecting an appropriate constraint-handling (CH) technique for a practical problem with varying conditions is a complex challenge. Determining the level of non-linearity in the objective functions, understanding the constraint, and identifying boundaries of the solution space is not always easy to identify or define. Therefore, it is desirable for evaluating multiple CH techniques to identify the most effective approach for a given problem.

### Test system I: IEEE 30 bus

Table 7 presents the configuration of all control and state variables, along with their permissible limits. This aligns with the best fitness achieved of the objective function using the IEEE 30 bus system. This analysis is conducted using one of the three CH techniques: ECO, SFS and SRA. The real power produced by the slack generator, along with the reactive power generated by all units, are treated as state variables. These are then included as constraints within the optimisation framework. The values provided in the table demonstrate that the constraint-handling methods effectively adhere to the specified boundaries of these constrained parameters. The permissible boundaries for generator reactive power are taken employing MATPOWER^34^. Additionally, the final row of the table specifies the approximate computational time required for a single execution of the case study on the processor (CPU). It is important to highlight that, due to the stochastic nature of population-based optimization algorithms, different executions may yield slightly varying optimal objective values. However, the decision variables consistently fall within comparable ranges, and the convergence behaviour remains similar across different runs.

**Table 7:** Optimized simulation results for effective 3-CH solutions in IEEE 30 Bus.

Parameters	Unit	Range^34^	Case 1	Case 2	Case 3	Case 4	Case 5	Case 6
CH			ECO	ECO	SFS	SFS	SFS	SFS
P_G1_	MW	50–200	168.2556	140.1962	80.3199	63.1537	51.5956	193.3564
P_G2_	MW	20–80	50.6114	54.9892	80.0012	68.4608	79.9999	49.5522
P_G5_	MW	15–50	22.2578	24.0374	50.0164	49.9881	49.9881	18.9668
P_G8_	MW	10–35	25.3464	34.9898	35.8836	34.8976	34.8976	10.0001
P_G11_	MW	10–30	12.7455	17.5662	29.8913	30.0021	30.0021	9.9999
P_G13_	MW	12–40	13.2056	18.3422	11.8438	39.9999	39.9999	11.9999
V_1_	p.u.	0.95–1.10	1.0738	1.0658	1.0414	1.0522	1.0510	1.0726
V_2_	p.u.	0.95–1.10	1.0543	1.0506	1.0372	1.0461	1.0468	1.0508
V_5_	p.u.	0.95–1.10	1.0227	1.0221	1.0602	1.0266	1.0272	1.0180
V_8_	p.u.	0.95–1.10	1.0273	1.0305	1.0445	1.0331	1.0335	1.0250
V_11_	p.u.	0.95–1.10	1.0854	1.0712	1.0883	1.0611	1.0751	1.0852
V_13_	p.u.	0.95–1.10	1.0321	1.0363	1.0814	1.0412	1.0408	1.0483
Q_C10_	MVAr	0–5	1.4568	4.6238	4.8873	4.4873	0.0023	4.8762
Q_C12_	MVAr	0–5	4.8182	3.0342	0.0036	4.4042	4.7685	0.5028
Q_C15_	MVAr	0–5	4.1382	4.4083	0.0127	4.2237	4.3425	3.8215
Q_C17_	MVAr	0–5	5	4.9965	0.7326	4.9999	4.9998	5
Q_C20_	MVAr	0–5	3.8365	3.7623	0.2354	3.7637	3.8834	4.1324
Q_C21_	MVAr	0–5	5	5	0	5	5	5
Q_C23_	MVAr	0–5	2.7628	2.4824	0.0182	3.1623	2.8854	3.0852
Q_C24_	MVAr	0–5	5	4.9995	0.0038	5	5	5
Q_C29_	MVAr	0–5	2.3564	2.2874	0.0001	2.2421	2.2542	2.3785
T_11_	p.u.	0.90–1.10	1.0433	1.0741	1.0341	1.0777	1.0726	1.0180
T_12_	p.u.	0.90–1.10	0.9228	0.9136	0.9101	0.9100	0.9020	1.0005
T_15_	p.u.	0.90–1.10	0.9576	0.9674	1.0224	0.9948	0.9971	0.9783
T_36_	p.u.	0.90–1.10	0.9652	0.9626	0.9521	0.9657	0.9662	0.9662
Q_G1_	MVAr	[−20–150]	6.4312	2.6641	−19.6521	−1.7512	−2.4716	6.2833
Q_G2_	MVAr	[−20–60]	25.5332	20.2212	−19.8585	12.2110	12.2157	20.2842
Q_G5_	MVAr	[−15–62.5]	27.5087	26.7882	60.4135	23.6228	23.6668	26.2287
Q_G8_	MVAr	[−15–48.7]	29.1281	29.4832	48.5863	29.2129	29.3622	29.4513
Q_G11_	MVAr	[−10–40]	28.0714	25.1522	26.8351	23.0312	27.8423	24.3712
Q_G13_	MVAr	[−15–44.7]	−6.5058	−1.6114	33.1715	2.5186	2.3248	7.2801
Cost	$/h		**800.4129**	**646.1331**	919.4142	944.2973	967.5523	**832.0691**
Emission	t/h		0.36510	0.27240	0.25320	**0.20481**	0.20812	0.42173
P_Loss_	MW		9.0223	6.72100	4.5562	3.1022	**3.08321**	10.4753
CVD	p.u.		0.8972	0.91130	0.9021	0.89210	0.8821	0.86960
VSI			0.13727	0.13712	**0.13627**	0.13860	0.13871	0.13915
CPU time	s		153.7	150.6	154.5	153.4	152.6	148.8

Table 8 presents a comparative analysis of the results obtained using the three CH techniques in this study alongside those from previous research.

In Case 1, which focuses on optimising the basic cost, the QPSODM-ECO and QPSODM-SFS algorithms yield fuel cost of **800.4129 $/h** and **800.4130 $/h**, respectively, while adhering to the system constraint. Notably, these results respect critical inequality constraint, including generators reactive power limits, loadbus voltage regulations and line capability. Considering these constraints, the voltage limits at load buses are particularly crucial, as their operating voltages frequently approach permissible limits. Several recent studies have reported better fuel cost results compared to those obtained using the three CH methods in this paper. However, a closer examination of these findings reveals instances of voltage limit violations [0.95–1.05] p.u., which render the solutions infeasible.

**Table 8:** Comparison of 3-CH methods with previous works - IEEE 30-Bus.

Case	Algorithm	Cost ($/h)	Emission (t/h)	*P*_Loss_ (MW)	CVD (p.u.)	VSI
Case 1	QPSODM-ECO	**800.4129**	0.36510	9.0223	0.8972	0.13727
	QPSODM-SFS	800.4130	0.36614	9.0286	0.91286	0.13804
	QPSODM-SRA	800.5121	0.36614	9.0263	0.89480	0.13803
	ACDE-SF^1^	800.41132	0.36634	9.00447	0.92279	0.13779
	EWOA^51^^b^	726.7182	0.2264	NR	NR	NR
	GA-GSF^52^^b^	796.2202	NR	8.3831a	NR	NR
	ECHT-DE^22^	800.4148	0.36593	8.9999	0.91231	0.13777
	KOA^53^	800.5512	0.3660	9.0319	0.9147	NR
	RTH^54^^b^	799.0680	NR	NR	NR	NR
	CBO^55^	832.0974	0.3195	10.68265	0.864783	NR
	ISSA^56^	800.4752	NR	9.1044	0.8965	0.1279
	WSO^14^^b^	781.733	1.7632	5.80114	0.468569	NR
	SMA^8^	802.5449	0.3635	9.5232	NR	NR
	C2oDE-FR-ECM^25^	800.4117	0.36647	9.00730	0.923969	0.13789
	BSA^6^^a^	799.0760	0.3671	8.6543	1.9129	0.1273
	ICBO^7^^a^	799.0353	NR	8.6132	1.9652	0.1261
Case 2	QPSODM-ECO	**646.1331**	0.27240	6.72100	0.91130	0.13712
	QPSODM-SFS	646.5882	0.28341	6.7381	0.92121	0.13782
	QPSODM-SRA	646.7373	0.28337	6.7528	0.91618	0.13803
	ECHT-DE^22^	646.4532	0.28351	6.7220	0.92671	0.13812
	ISSA^56^	646.1336	NR	6.6717	0.6257	0.1343
	C2oDE-FR-ECM^25^	646.40372	0.283529	6.71765	0.93575	0.13768
Case 3	QPSODM-ECO	880.1420	0.27314	5.9213	0.87913	0.13633
	QPSODM-SFS	919.4142	0.25320	4.5562	0.9021	**0.13627**
	QPSODM-SRA	819.1205	0.27325	6.6871	0.82284	0.13742
	ACDE-SF^1^	919.70514	0.210553	3.994983	0.911914	0.136447
	ECHT-DE^22^	917.5916	0.2252	4.5224	0.9110	0.13632
	RTH^54^^b^	799.4000	NR	NR	NR	0.1134
	FDBAGDE^57^^b^	905.8749	0.1096	3.2581	0.8898	0.13787
	C2oDE-FR-ECM^25^	920.25346	0.2253	4.5049	0.9004	0.136283
Case 4	QPSODM-ECO	944.3877	**0.20481**	3.2332	0.88713	0.13856
	QPSODM-SFS	944.2973	**0.20481**	3.1022	0.89210	0.13860
	QPSODM-SRA	944.3842	**0.20481**	3.2347	0.88372	0.13868
	ACDE-SF^1^	944.3269	0.204817	3.21683	0.89909	0.138287
	EWOA^51^^b^	734.5632	0.19730	NR	NR	NR
	ECHT-DE^22^	944.3782	0.20482	3.2179	0.89240	0.13844
	KOA^53^	944.4245	0.20480	3.2381	0.8874	NR
	SMA^8^	945.0454	0.20488	3.4375	NR	NR
	C2oDE-FR-ECM^25^	944.33192	0.204817	3.21678	0.902163	0.138235
Case 5	QPSODM-ECO	967.6624	0.20716	**3.08321**	0.89243	0.13844
	QPSODM-SFS	967.5523	0.20812	**3.08321**	0.8821	0.13871
	QPSODM-SRA	967.6158	0.20715	3.10091	0.88930	0.13857
	ACDE-SF^1^	967.62435	0.207265	3.084041	0.901717	0.138311
	ECHT-DE^22^	967.6001	0.20726	3.0850	0.90937	0.13836
	KOA^53^	967.6531	0.2073	3.1038	0.9098	NR
	RTH^54^^b^	NR	NR	2.8506	NR	NR
	FDBAGDE^57^^b^	861.0411	0.0993	2.0373	0.6235	0.1433
	CBO^55^^b^	832.1366	0.319488	3.0881	0.840011	NR
	SMA^8^	968.1335	0.207294	3.2975	NR	NR
	C2oDE-FR-ECM^25^	967.6239	0.20726	3.08391	0.90460	0.13816
Case 6	QPSODM-ECO	832.0709	0.42181	10.4711	0.84418	0.13917
	QPSODM-SFS	**832.0691**	0.42173	10.4753	0.86960	0.13915
	QPSODM-SRA	832.4142	0.42113	10.5273	0.55825	0.14248
	ACDE-SF^1^	832.07218	0.437578	10.65108	0.85271	0.13900
	ECHT-DE^22^	832.1356	0.43765	10.6772	0.80326	0.14039
	FDBAGDE^57^^b^	776.4826	1.7692	5.7028	0.7033	0.1414
	ISSA^56^	919.1915	NR	9.6737	0.6154	0.1325
	C2oDE-FR-ECM^25^	832.0708	0.43750	10.6468	0.84455	0.13912

^a^ Constraint at load bus voltage is violated (Infeasible solution).

^b^ A modified version was used (with integrated distributed generation (DG)…).

NR: Not reported in the literature.

**Fig. 6:**
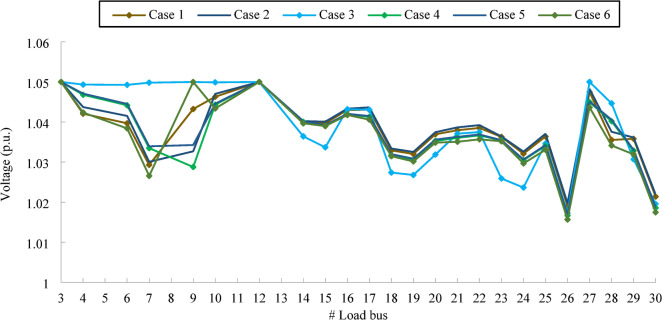
Voltage curves of load bus in IEEE 30-bus for the effective solutions.

A quick assessment of the voltage profiles in Fig. 6 suggests that these violations likely rise from exceeding the maximum voltage limit. Such overvoltage conditions are undesirable, as they can impose stress on the power system and potentially damage connected equipment. For the IEEE 30-bus analysed in this study, there are 24 load buses. In theory, if all the buses were to function precisely at their voltage limits, the total maximum absolute cumulative voltage deviation (CVD), as defined in Eq. (17), will be 1.20 p.u., that is, 24 multiplied by 0.05 (p.u.). However, reported CVD values in certain studies in BSA^6^, ICBO^7^ exceed this theoretical maximum, suggesting inconsistencies. This discrepancy is largely attributable to the use of the static penalty technique that is highly affected by the choice for the penalty factor.

In Table 8, such cases are explicitly marked with footnotes for clarity. In contrast, the CH techniques employed in this study ensure that all state and dependent variables remain within their prescribed limits, thereby maintaining solution feasibility. The static penalty function approach, on the other hand, frequently results in constraint violations, possibly due to an oversight in programming. A well-designed CH technique should be capable of achieving optimal results while operating close to the limits, without violating them, demonstrating its robustness and effectiveness in solving the OPF problem.

Figure 6 illustrates the voltage profiles of load buses for the IEEE 30 bus test system across different case studies. Each voltage profile corresponds to all control variables determined utilizing the most effective technique for that particular case, as outlined in Table 7. Notably, all optimal solutions remain within the prescribed voltage limits, ensuring compliance with system constraints.

In Case 2, which focuses on minimizing the cost of multiple fuel types, the QPSODM-ECO algorithm achieves an optimal cost of **646.1331 $/h**, demonstrating superior performance compared to other existing algorithms. Similarly, in Case 3, where the objective is to enhance voltage stability by minimizing the VSI of system load buses, QPSODM-SFS attains an optimal value of **0.13627**, exhibiting a slight improvement over comparable algorithms. The emission objective in (t/h) and the other relevant parameters attained using QPSODM-ECO, QPSODM-SFS, and QPSODM-SRA in Case 4 closely match those reported by the algorithm C2oDE-FR-ECM^25^, achieving an optimal value of **0.20481 t/h**.

Meanwhile, in Case 5, the QPSODM-ECO and QPSODM-SFS algorithms yield a power loss **3.08321 MW**, this is in strong agreement with the benchmark data presented in the corresponding comparison table.

Case 6 considers the valve-loading effect, which leads to a small increase cost than in Case 1, with QPSODM-SFS computing a final cost of **832.0691 $/h.**

**Fig. 7:**
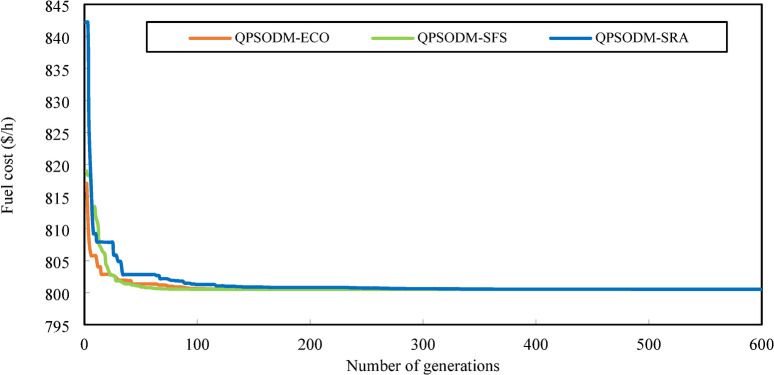
Comparison of convergence of the three CH methods - Case 1.

**Fig. 8:**
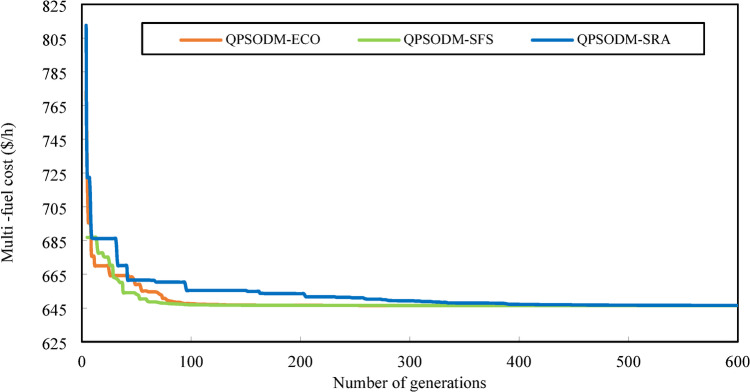
Comparison of convergence of the three CH methods - Case 2.

**Fig. 9:**
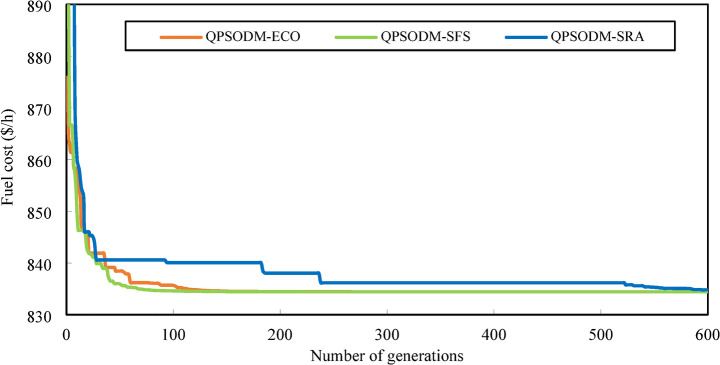
Comparison of convergence of the three CH methods - Case 6.

In summary, while performance variations are evident among the three CH methods, at least one of the proposed methods consistently ranks among the best-reported results for solving the OPF problem. However, rather than only aiming to achieve superior numerical results, the primary objective of this study is to ensure rigorous adherence to system constraints using CH-based methods. Figures 7 and 8, and Fig. 9 present a graphical comparison of the convergence curves of the three 3-CH methods: QPSODM-ECO, QPSODM-SFS, and QPSODM-SRA, across Case 1, Case 2, and Case 6 for cost-related function. The convergence speediness of QPSODM-ECO and QPSODM-SFS show no significant differences, as both algorithms demonstrate rapid and abrupt convergence during the initial stages of the search. In contrast, QPSODM-SRA approaches the optimal solution at a slower pace, often experiencing stagnation at intermediate points.

**Fig. 10:**
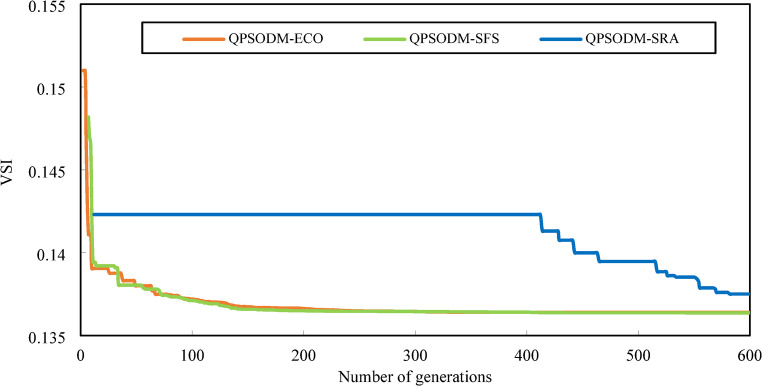
Comparison of convergence of the three CH methods - Case 3.

**Fig. 11:**
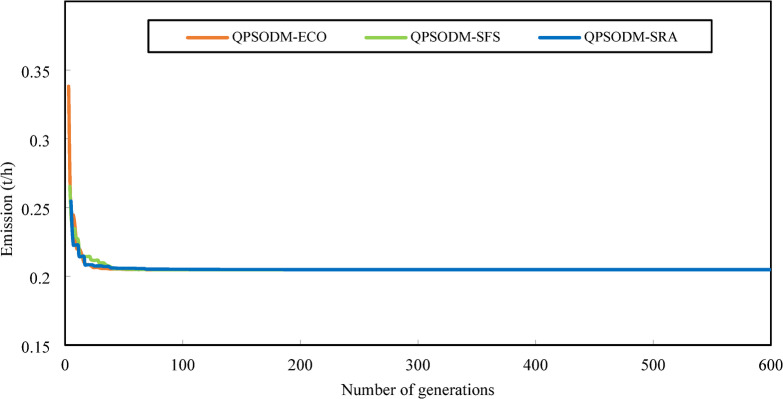
Comparison of convergence of the three CH methods - Case 4.

**Fig. 12:**
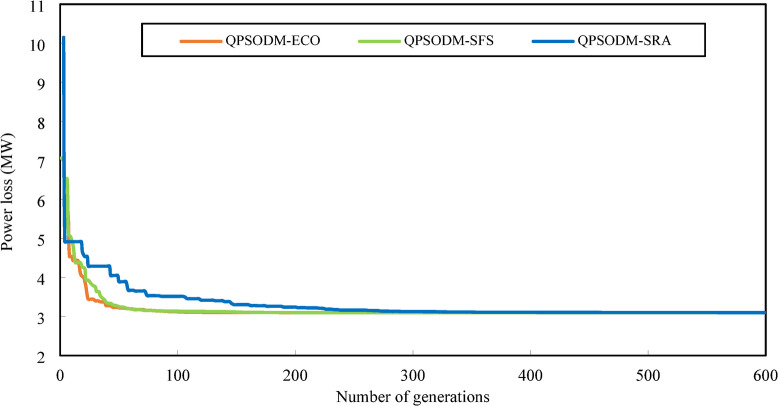
Comparison of convergence of the three CH methods - Case 5.

The convergence curves for Case 3, Case 4 and Case 5 are displayed in Figs. 10 and 11, and Fig. 12, respectively. In Case 3, the convergence behaviour of QPSODM-SRA appears somewhat unstable and exhibits sudden fluctuations, indicating an irregular optimization trajectory. The VSI objective function is strongly influenced by the most critical load bus voltage constraint, which presents a challenge for the SRA method in finding feasible solutions during the later phases of the search, thereby prolongs the optimization convergence. As a result, the convergence process is delayed. Overall, QPSODM-SFS and QPSODM-ECO consistently achieve optimal solutions within approximately 200 iterations.

### Test system II: IEEE 57 bus

The setting of dependent and independent variables, along with their respective intervals to achieve optimal fitness in all case studies related to the IEEE 57-bus test system, are presented in Table 9. Additionally, Table 10 provides a comparative analysis of the results obtained using the three constraint-handling (CH) methods: ECO, SFS and SRA against the latest studies available in the literature. The findings indicate that QPSODM-ECO consistently outperforms other methods in achieving the best fitness values, except in Case 7. Similar to test system I (IEEE 30-bus), all constraints are effectively managed through the implemented CH methods. The reactive power limits for generators, got from reference^34^, reveal relatively narrow margins for certain units. However, the CH methods ensure compliance with these constraints, as evidenced by the listed generator reactive power values. Moreover, the permitted voltage limit for load buses in the IEEE 57 bus is set as [0.94–1.06] p.u. In theory, if all the 50 loadbus were to function precisely at their voltage limits, the total maximum absolute cumulative voltage deviation (CVD), as defined in the Eq. (17), will be 3.00 p.u., that is, 50 multiplied by 0.06 per unit (p.u.). Notably, in one case, the reported CVD exceeds the theoretical limit. This deviation is specifically indicated in italics and highlighted with a footnote in Table 10 for clarity. The authors in^58^, associated with this case, employed a conventional static penalty function methodology to handle constraints.

**Table 9:** Optimized simulation results for effective 3-CH solutions in IEEE 57 Bus.

Parameters	Range^34^	Case 7	Case 8	Case 9	Parameters	Range	Case 7	Case 8	Case 9
CH		SFS	ECO	ECO	CH		SFS	ECO	ECO
P_G1_ (MW)	0–576	142.8120	307.5354	178.8455	T_46_ (p.u.)	0.90–1.10	0.9618	0.9179	0.9554
P_G2_ (MW)	30–100	89.2822	35.1532	29.9999	T_54_ (p.u.)	0.90–1.10	0.9132	0.9001	0.9145
P_G3_ (MW)	40–140	44.1021	127.7215	133.3875	T_58_ (p.u.)	0.90–1.10	0.9813	0.9287	0.9886
P_G6_ (MW)	30–100	71.4487	30.8415	100.0011	T_59_ (p.u.)	0.90–1.10	0.9661	0.9888	0.9734
P_G8_ (MW)	100–550	461.3587	285.1467	307.2902	T_65_ (p.u.)	0.90–1.10	0.9761	1.0129	0.9799
P_G9_ (MW)	30–100	97.3271	100.0002	100.0021	T_66_ (p.u.)	0.90–1.10	0.9375	0.9001	0.9441
P_G12_ (MW)	100–410	359.3213	382.1628	411.1443	T_71_ (p.u.)	0.90–1.10	0.9751	0.9691	0.9782
V_G1_ (p.u.)	0.95–1.10	1.0556	1.0027	1.0612	T_73_ (p.u.)	0.90–1.10	0.9927	1.0038	0.9922
V_G2_ (p.u.)	0.95–1.10	1.0633	1.0021	1.0562	T_76_ (p.u.)	0.90–1.10	0.9659	0.9002	0.9633
V_G3_ (p.u.)	0.95–1.10	1.0452	1.0125	1.0556	T_80_ (p.u.)	0.90–1.10	1.0047	0.9941	0.9933
V_G6_ (p.u.)	0.95–1.10	1.0623	1.0021	1.0531	Q_G1_ (MW)	[−140–200]	45.9823	−49.5433	42.2936
V_G8_(p.u.)	0.95–1.10	1.0651	1.0227	1.0601	Q_G2_ (MW)	[−17–50]	49.9955	49.9832	49.9963
V_G9_ (p.u.)	0.95–1.10	1.0407	1.0132	1.0413	Q_G3_ (MW)	[−10–60]	34.6592	59.9963	34.0155
V_G12_ (p.u.)	0.95–1.10	1.0427	1.0313	1.0463	Q_G6_ (MW)	[−8–25]	−7.9448	−7.5132	−7.3511
Q_C18_ (MVAr)	0–20	6.3727	0.0262	0.4977	Q_G8_ (MW)	[−140–200]	53.5055	44.3912	48.1222
Q_C25_ (MVAr)	0–20	12.0663	20.0005	14.4213	Q_G9_ (MW)	[−3–9]	8.9946	8.9970	8.9951
Q_C53_ (MVAr)	0–20	11.5318	20.0004	13.1603	Q_G12_ (MW)	[−150–155]	57.0852	154.6523	48.7111
T_19_ (p.u.)	0.90–1.10	0.9687	1.0995	1.0603	P_Loss_ (MW)		14.8521	17.7613	**9.8706**
T_20_ (p.u.)	0.90–1.10	1.0017	0.9082	0.9241	CVD (p.u.)		1.6958	**0.5835**	1.8016
T_31_ (p.u.)	0.90–1.10	1.0082	0.9689	1.0091	VSI		0.2773	0.3024	0.2771
T_35_ (p.u.)	0.90–1.10	0.9981	1.0489	1.0889	Emission (t/h)		1.3524	1.2981	1.1037
T_36_ (p.u.)	0.90–1.10	1.0101	1.0918	0.9473	Cost ($/h)		**41665.78**	46712.52	44560.51
T_37_ (p.u.)	0.90–1.10	1.0344	1.0063	1.0112	CPU (s)		225.8	214.5	212.3
T_41_ (p.u.)	0.90–1.10	0.9947	0.9969	0.9958					

**Table 10:** Comparison of 3-CH methods with previous works - IEEE 57-Bus.

Case	Algorithm	Cost ($/h)	Emission (t/h)	*P*_Loss_ (MW)	CVD (p.u.)	VSI
Case 7	QPSODM-ECO	41666.12	1.3541	14.8540	1.6937	0.2778
	QPSODM-SFS	**41665.78**	1.3524	14.8521	1.6958	0.2773
	QPSODM-SRA	41670.87	1.3504	14.9912	1.5281	0.2811
	APFPA^58^^a^	41628.75	NR	14.0470	*3.5571*	NR
	GA-GSF^52^^b^	41570.56	NR	68.2536	NR	NR
	EWOA^51^^b^	35156.00	1.074	NR	NR	NR
	CBO^55^	41666.24	1.3524	14.8596	1.6973	0.2786
	ISSA^56^	41675.02	NR	14.5290	0.9847	0.24095
	SMA^8^	41697.11	1.9332	15.5557	NR	NR
	C2oDE-FR-ECM^25^	41666.24	1.35436	14.8698	1.71752	0.278628
	ECHT-DE^22^	41670.56	1.36230	14.9479	1.50319	0.28886
Case 8	QPSODM-ECO	46712.52	1.2981	17.7613	**0.5835**	0.3024
	QPSODM-SFS	46224.37	1.2689	15.5535	0.5849	0.3025
	QPSODM-SRA	44437.67	1.2041	14.7121	0.6012	0.3012
	KOA^53^	50223.38	1.5334	26.3651	0.5836	NR
	C2oDE-FR-ECM^25^	43805.62	1.15070	15.7835	0.58568	0.30146
	ECHT-DE^22^	46813.22	1.3379	19.0821	0.60416	0.3008
	SF-DE^22^	45246.02	1.23453	18.4697	0.59584	0.30135
	SP-DE^22^	45549.49	1.2898	18.4275	0.59267	0.30052
Case 9	QPSODM-ECO	44560.51	1.1037	**9.8706**	1.8016	0.2771
	QPSODM-SFS	44503.41	1.1052	9.8742	1.7771	0.2774
	QPSODM-SRA	44566.51	1.1042	9.9716	1.5926	0.2834
	CBO^55^	44567.04	1.10504	9.95571	1.67981	0.27913
	SMA^8^	45,049.13	1.4032	10.6734	NR	NR

^a^ Constraint at load bus voltage is violated (Infeasible solution).

^b^ A modified version was used (with integrated DG…).

NR: Not reported in the literature.

In Case 7, where the primary objective is to minimize fuel cost, the QPSODM-SFS algorithm achieves the lowest recorded value of **41665.78 $/h**, outperforming other recent studies, as shown in Table 9. Additionally, the power loss is significantly low at **14.8521 MW**, demonstrating the effectiveness of the recommended control variable settings used by QPSODM-SFS.

Case 8 focuses on optimization aimed at reducing cumulative voltage deviation. The results obtained using any of the three applied methods show substantial improvement compared to the other algorithms, albeit at the expense of higher fuel costs. The increase in cost is attributed to the recommended control variable settings.

**Fig. 13:**
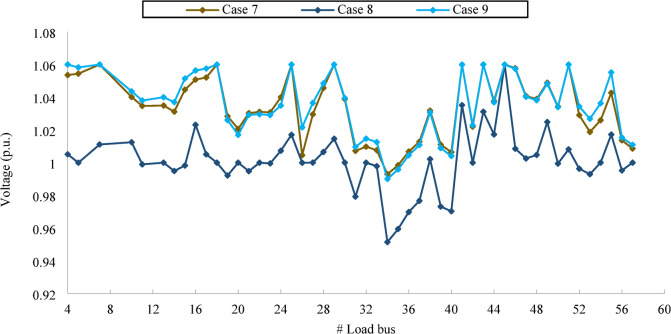
Voltage profiles of load bus in IEEE 57-bus for the effective solutions.

In Case 9, which targets the minimization of the power loss, the QPSODM-EC algorithm achieves the lowest recorded value of **9.8706 MW**. Overall, the fitness values obtained across all cases using the three applied methods demonstrate superior performance when compared to most existing studies in the literature. The voltage curves of all load bus corresponding to the optimal solutions obtained in the IEEE 57 bus test case studies are illustrated in Fig. 13. It is evident that all bus voltages remain within permissible limits.

However, in certain cases, a significant number of buses voltage is observed to be near to the superior bound, highlighting the necessity for meticulous attention in enforcing voltage constraints to prevent overvoltage occurrences.

**Fig. 14:**
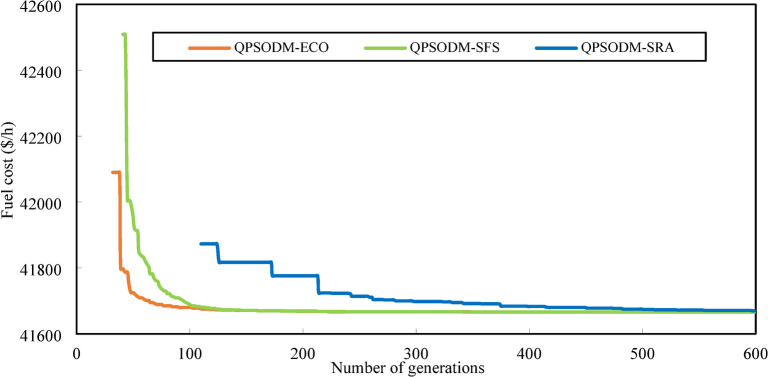
Comparison of convergence of the three CH methods - Case 7.

Figure 14 presents the convergence curves for Case 7, where the application of 3-CH methods demonstrates a relatively rapid convergence toward the fitness value for both QPSODM-ECO and QPSODM-SFS. Notably, QPSODM-ECO shows a faster convergence compared to the other methods. The plotted curves commence after a predefined number of generations, omitting the initial search phase.

It is important to note that the CH method primarily seeks feasible solutions in the early stages of the optimization course. The process of converging to the optimal solution truly starts once the search has effectively entered the feasible region. Concerning the IEEE 30 bus test system, the employed constraint-handling methods simply identify the solution space. However, in the IEEE 57-bus system, the narrow permissible range for generator reactive power imposes additional challenges, often requiring more generations before reaching feasibility. The initial, inconsistent phase of the search is not captured in the convergence curves.

**Fig. 15:**
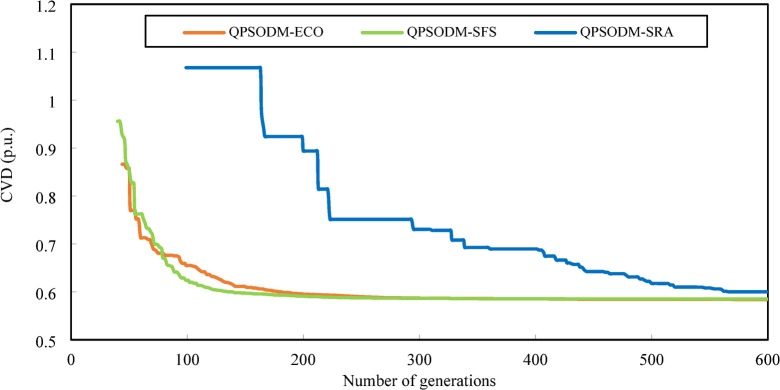
Comparison of convergence of the three CH methods - Case 8.

**Fig. 16:**
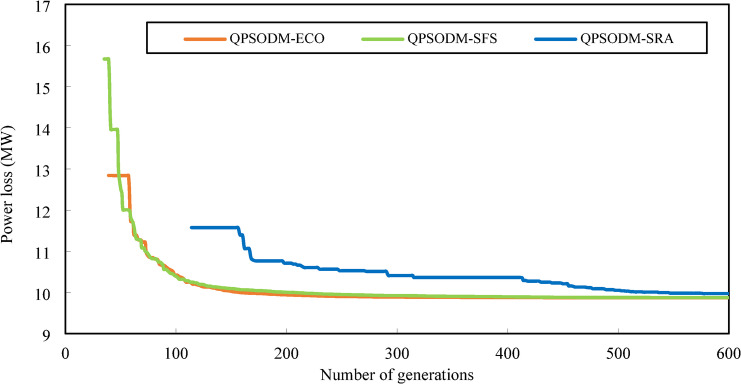
Comparison of convergence of the three CH methods - Case 9.

Figure 15 depicts the convergence curves for Case 8, where the QPSODM-SRA algorithm shows notably slow convergence, necessitating a significantly higher number of generations to achieve an optimal solution. Additionally, Fig. 16 presents a comparative analysis of the convergence curve for Case 9, focusing on minimizing power loss. Generally, QPSODM-SRA demands more generations than the other methods, both in finding feasible solutions and in achieving final convergence to optimality.

### Test system III: IEEE 118-bus

To evaluate the performance of the proposed approach under large-scale non-linear OPF conditions, the IEEE 118-bus test system has been selected, incorporating valve-point effects, multi-fuel cost functions, and a high-dimensional control space. Although larger benchmark systems exist in platforms such as MATPOWER, the IEEE 118-bus system is widely recognized as a large-scale benchmark when realistic non-linearities and complex constraint interactions are considered, making it suitable for assessing algorithmic robustness and scalability. As reported in Table 5, the QPSODM-ECO variant demonstrates superior performance in handling the IEEE 118-bus system, which involves a significantly larger number of control variables and operational constraints compared to smaller test systems. Specifically, the system includes 130 control variables, resulting in a high-dimensional and tightly constrained optimization problem. For this case study, a population size of 100 and a maximum of 2000 iterations were employed, leading to an execution time of approximately 28 min. The detailed control variables and corresponding optimal fitness values obtained for Case 10 are presented in Table 11. The allowable limits for generator real power are taken from^34^, while shunt capacitor ratings are varied between 0 and 25 MVAr. Generator voltage magnitudes and transformer Tap settings are constrained within [0.95–1.10] p.u. and [0.90–1.10] p.u., respectively.

**Table 11:** Results of QPSODM-ECO for the Case 10 of the IEEE 118-bus test.

Parameter	Range [MW] [34]	Value [MW]	Parameter	Range [MW] [34]	Value [MW]	Parameter	Value [p.u.]	Parameter	Value [p.u.]	Parameter	Value
P_G1_	[30–100]	30.4140	P_G65_	[148–491]	288.675	V_G1_	1.032	V_G65_	1.0622	Q_C5_ (MVAr)	4.098
P_G4_	[30–100]	30.0022	P_G66_	[148–492]	287.038	V_G4_	1.0578	V_G66_	1.0735	Q_C34_ (MVAr)	4.2657
P_G6_	[30–100]	30.0005	P_G69_	[0–805]	368.916	V_G6_	1.0513	V_G69_	1.0723	Q_C37_ (MVAr)	0
P_G8_	[30–100]	29.9899	P_G70_	[30–100]	28.8903	V_G8_	1.0379	V_G70_	1.0563	Q_C44_ (MVAr)	3.5564
P_G10_	[165–550]	326.5852	P_G72_	[30–100]	28.889	V_G10_	1.0488	V_G72_	1.0613	Q_C45_ (MVAr)	18.7873
P_G12_	[56–185]	68.2275	P_G73_	[30–100]	28.8919	V_G12_	1.0464	V_G73_	1.0596	Q_C46_ (MVAr)	12.3428
P_G15_	[30–100]	29.9999	P_G74_	[30–100]	28.8914	V_G15_	1.046	V_G74_	1.0461	Q_C48_ (MVAr)	6.9565
P_G18_	[30–100]	29.9999	P_G76_	[30–100]	28.889	V_G18_	1.0484	V_G76_	1.0266	Q_C74_ (MVAr)	24.7828
P_G19_	[30–100]	29.9999	P_G77_	[30–100]	28.8891	V_G19_	1.0459	V_G77_	1.0511	Q_C79_ (MVAr)	24.867
P_G24_	[30–100]	30.0002	P_G80_	[173–577]	347.681	V_G24_	1.0572	V_G80_	1.0593	Q_C82_ (MVAr)	24.8882
P_G25_	[96–320]	160.8914	P_G85_	[30–100]	28.8924	V_G25_	1.0712	V_G85_	1.0591	Q_C83_ (MVAr)	11.9794
P_G26_	[124–414]	229.9536	P_G87_	[31–104]	30.089	V_G26_	1.0782	V_G87_	1.0746	Q_C105_ (MVAr)	3.8059
P_G27_	[30–100]	30.0011	P_G89_	[212–707]	383.341	V_G27_	1.0497	V_G89_	1.0711	Q_C107_ (MVAr)	24.6909
P_G31_	[32–107]	32.1302	P_G90_	[30–100]	28.889	V_G31_	1.0456	V_G90_	1.0568	Q_C110_ (MVAr)	0
P_G32_	[30–100]	30.0007	P_G91_	[30–100]	28.889	V_G32_	1.048	V_G91_	1.0632	T_8_ (p.u.)	0.9756
P_G34_	[30–100]	30.0007	P_G92_	[30–100]	28.8946	V_G34_	1.0543	V_G92_	1.062	T_32_ (p.u.)	1.0588
P_G36_	[30–100]	30	P_G99_	[30–100]	28.8891	V_G36_	1.0515	V_G99_	1.0618	T_36_ (p.u.)	0.9823
P_G40_	[30–100]	30.0003	P_G100_	[106–352]	175.974	V_G40_	1.0369	V_G100_	1.0647	T_51_ (p.u.)	0.9772
P_G42_	[30–100]	30.0038	P_G103_	[42–140]	40.8892	V_G42_	1.0386	V_G103_	1.0628	T_93_ (p.u.)	0.9805
P_G46_	[36–119]	35.7001	P_G104_	[30–100]	28.889	V_G46_	1.0545	V_G104_	1.058	T_95_ (p.u.)	0.9978
P_G49_	[91–304]	161.4982	P_G105_	[30–100]	28.889	V_G49_	1.063	V_G105_	1.0558	T_102_ (p.u.)	0.9698
P_G54_	[44–148]	44.4082	P_G107_	[30–100]	28.8891	V_G54_	1.0455	V_G107_	1.0494	T_107_ (p.u.)	0.9423
P_G55_	[30–100]	30.0001	P_G110_	[30–100]	28.889	V_G55_	1.0457	V_G110_	1.0592	T_127_ (p.u.)	0.9829
P_G56_	[30–100]	30.0002	P_G111_	[41–136]	39.6891	V_G56_	1.0452	V_G111_	1.0687	Cost ($/h)	127895.84
P_G59_	[77–255]	124.6466	P_G112_	[30–100]	28.889	V_G59_	1.0617	V_G112_	1.0509	P_loss_ (MW)	57.7743
P_G61_	[78–260]	123.0044	P_G113_	[30–100]	28.889	V_G61_	1.0604	V_G113_	1.0554	CVD (p.u.)	3.09858
P_G62_	[30–100]	30.003	P_G116_	[30–100]	28.8894	V_G62_	1.0542	V_G116_	1.0601	VSI	0.06159

**Fig. 17:**
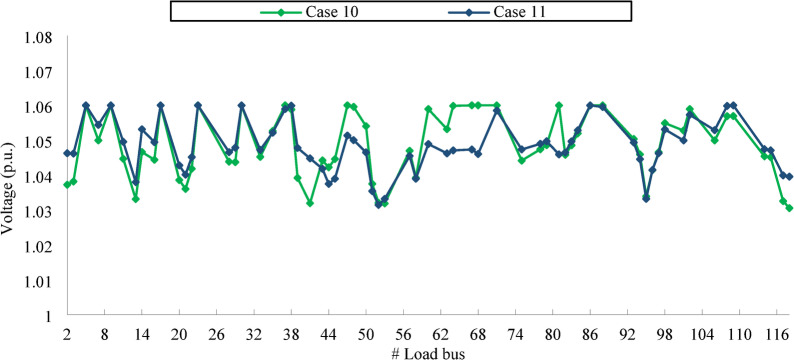
Voltage profiles of load bus in IEEE 118-bus for the effective solutions.

**Fig. 18:**
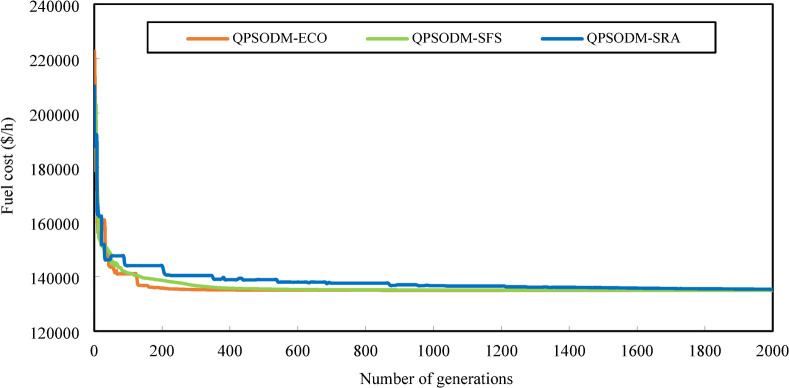
Comparison of convergence of the three CH methods - Case 10.

**Fig. 19:**
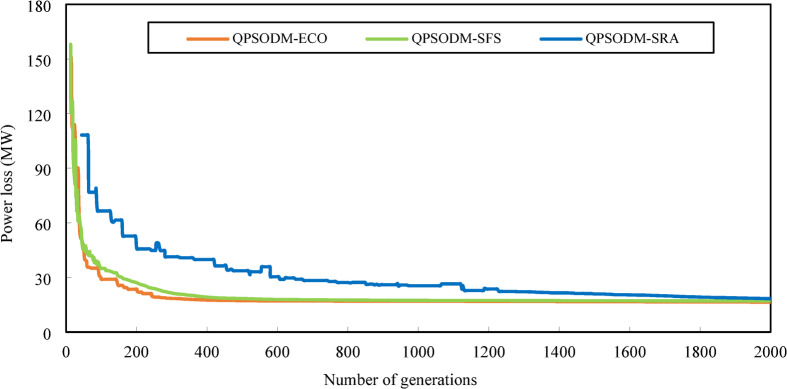
Comparison of convergence of the three CH methods - Case 11.

The voltage curves for the loadbus in both cases are illustrated in Fig. 17. Whereas the comparison of the convergence of the 3-CH methods for Case 10 and Case 11 is depicted in Figs. 18 and 19, respectively.

Due to the extensive number of constraints, the algorithm requires approximately 300 generations to identify the initial solution space. The algorithm demonstrates clear signs of convergence toward optimality after approximately 1500 generations, suggesting stabilization in the search process.

Additionally, Table 12 provides a comparative analysis of the results obtained using the three CH methods against the recent studies.

In Case 10, the optimal fuel cost is determined to be **127895.84 $/h**, closely aligning with the results reported in^8^. In Case 11, the power loss is effectively minimized to **16.46353 MW**. Notably, both cases achieved their best performance using the QPSODM-ECO algorithm, demonstrating its effectiveness in optimizing fuel cost and reducing power loss. Given the complexity of the system, which includes a significant number of control variables and constraints, the algorithm effectively manages up to 118 critical constraints related to generator reactive power and load buses. It successfully satisfies all imposed constraints, ensuring robust performance.

**Table 12:** Comparison of 3-CH methods with previous works - IEEE 118-Bus.

Case	Algorithm	Cost ($/h)	Emission (t/h)	*P*_Loss_ (MW)	CVD (p.u.)	VSI
Case 10	QPSODM-ECO	**127895.84**	8.5091	57.77432	3.09858	0.061596
	QPSODM-SFS	129658.64	8.6182	58.74255	2.66126	0.062737
	QPSODM-SRA	135350.32	8.9987	64.23950	1.26801	0.063491
	EWOA^51^^a, b^	127275.00	NR	43.9700	NR	NR
	KOA^53^	135074.35	NR	60.2110	2.2259	NR
	ISSA^56^	129460.83	NR	NR	1.0529	0.0624
	SMA^8^	127896.54	8.4962	79.4121	NR	NR
	C2oDE-FR-ECM^25^	134943.80	NR	58.2061	NR	NR
	SP-DE^22^	135055.70	NR	NR	NR	NR
Case 11	QPSODM-ECO	155218.95	6.5963	**16.46353**	2.62274	0.06305
	QPSODM-SFS	155383.53	6.4592	17.06636	1.32842	0.06656
	QPSODM-SRA	153955.68	6.8546	18.38184	0.97750	0.06590
	EWOA^51^ ^a, b^	131573.00	NR	27.9300	NR	NR
	KOA^53^	155565.44	NR	17.7949	1.8401	NR
	SP-DE^22^	NR	NR	17.6946	NR	NR

^a^ Constraint at load bus voltage is violated (Infeasible solution).

^b^ A modified version was used (with integrated DG…).

NR: Not reported in the literature.

### Discussion on the impact of CH techniques

The results confirm that the choice of CH technique significantly influences the performance of QPSODM in OPF problems. While QPSODM-SFS and QPSODM-ECO show comparable behavior in several cases, QPSODM-ECO consistently delivers superior or more robust solutions, particularly for large-scale and highly constrained systems. In contrast, QPSODM-SRA generally exhibits weaker performance. The superior performance of the ε-constraint approach (ECO) stems from its adaptive feasibility relaxation mechanism. Unlike SFS, which strictly enforces feasibility and may prematurely discard promising infeasible solutions, ECO enables controlled exploration of infeasible regions through a gradually decreasing ε parameter. This feature is especially beneficial for OPF problems, where feasible regions are often narrow and located near constraint boundaries. This adaptive behavior aligns well with the quantum-behaved dynamics of QPSODM. The stochastic position updates and dynamic *Mbest* promote broad exploration, which is not prematurely restricted when combined with ECO, allowing the algorithm to progressively converge toward high-quality feasible solutions. This synergy explains the improved scalability and robustness of QPSODM-ECO in larger systems. In contrast, SFS may suffer from reduced population diversity due to strict feasibility enforcement, leading to stagnation in complex cases. This trend is supported by the statistical analysis, where QPSODM-ECO exhibits medium to large effect sizes over QPSODM-SFS. The SRA shows limited effectiveness, as its probabilistic balance between objective values and constraint violations can result in unstable selection pressure. Despite its advantages, the ε-constraint method remains sensitive to parameter settings and may bias the search toward constraint boundaries if not carefully tuned. Nonetheless, under the adopted configuration, ECO achieves a favorable balance between exploration and feasibility enforcement, making it particularly effective for large-scale and tightly constrained OPF problems.

## Conclusions

This study evaluated the effectiveness of three constraint-handling (CH) techniques: Epsilon ($\epsilon$) constraint (ECO), Superiority of feasible solutions (SFS), and Stochastic ranking (SRA), when integrated with the QPSODM algorithm for solving OPF problems. The assessment was carried out across multiple test systems using numerical performance indicators and the Wilcoxon signed-rank test complemented by rank-biserial correlation effect size analysis. The results show that although no single CH method dominates in all cases, QPSODM-ECO achieves superior or statistically equivalent performance in the majority of scenarios. Specifically, ECO outperformed SFS in Case 1, Case 8, and Case 10, with medium-to-large effect sizes, while both methods exhibited statistically similar behavior in most remaining cases. In contrast, QPSODM-SRA was consistently outperformed by both ECO and SFS, except in Case 4, where all three methods yielded comparable results. Numerical outcomes further indicate a consistent reduction in hourly operational cost across most test cases, confirming the economic efficiency of the proposed approach. These findings highlight the critical role of effective constraint handling in OPF optimization. Inadequate constraint management may lead to boundary violations, increased losses, or degraded feasibility, whereas the ε-constraint mechanism effectively balances feasibility enforcement and search diversity, particularly in large-scale systems with many decision variables. Overall, the QPSODM algorithm combined with the ε-constraint approach demonstrates strong robustness, scalability, and suitability for complex OPF applications, supported by both numerical improvements and statistical evidence.

### Future work

Future research may extend this work in several directions. First, the proposed framework can be applied to larger and more diverse benchmark systems to further assess scalability and computational efficiency. Second, multi-objective OPF formulations incorporating economic, environmental, and voltage stability objectives could be explored to better reflect real-world operational requirements. Additionally, adaptive or hybrid constraint-handling strategies that dynamically adjust parameters during the optimization process may further enhance convergence speed and feasibility robustness. Finally, incorporating renewable energy uncertainty, variable demand, and contingency (*N* − 1) analysis would provide a more comprehensive evaluation of the proposed approach under realistic operating conditions.

## Data Availability

The data used and/or analyzed during the current study are available from the corresponding author upon reasonable request.

## Appendix

**Table 13:** Generator cost and emission parameters - IEEE 30-bus [6].

# Bus	1	2	5	8	11	13
# Generator	G_1_	G_2_	G_5_	G_8_	G_11_	G_13_
*a *($/h)	0	0	0	0	0	0
*b *($/MWh)	2	1.75	1	3.25	3	3
*c *($/MW^2^h)	0.00375	0.0175	0.0625	0.00834	0.025	0.025
*d *($/h)	18	16	14	12	13	13.5
*e *(rad/MW)	0.037	0.038	0.04	0.045	0.042	0.041
*α*	4.091	2.543	4.258	5.326	4.258	6.131
*β*	-5.554	-6.047	-5.094	-3.55	-5.094	-5.555
*γ*	6.49	5.638	4.586	3.38	4.586	5.151
*ω*	0.0002	0.0005	0.000001	0.002	0.000001	0.00001
*µ*	6.667	3.333	8	2	8	6.667

**Table 14:** Generator cost and emission parameters - IEEE 57-bus [6].

# Bus	1	2	3	6	8	9	12
# Generator	G_1_	G_2_	G_3_	G_6_	G_8_	G_9_	G_12_
*a *($/h)	0	0	0	0	0	0	0
*b *($/MWh)	20	40	20	40	20	40	20
*c *($/MW^2^h)	0.0775795	0.01	0.25	0.01	0.0222222	0.01	0.0322581
*d *($/h)	18	16	13.5	18	14	15	12
*e *(rad/MW)	0.037	0.038	0.041	0.037	0.04	0.039	0.045
*α*	4.091	2.543	6.131	3.491	4.258	2.754	5.326
*β*	-5.554	-6.047	-5.555	-5.754	-5.094	-5.847	-3.555
*γ*	6.49	5.638	5.151	6.39	4.586	5.238	3.38
*ω*	0.0002	0.0005	0.00001	0.0003	0.000001	0.0004	0.002
*μ*	0.286	0.333	0.667	0.266	0.8	0.288	0.2

**Table 15:** Cost coefficients for the generators 1 and 2 considering multi-fuel sources - IEEE 30 bus [6].

# Bus	# Generator	*a*	*b*	*c*	$P_{g1}^{min}$	$P_{g1}^{max}$	*a*	*b*	*c*	$P_{g2}^{min}$	$P_{g2}^{max}$
1	G_1_	55	0.7	0.005	50	140	82.5	1.05	0.0075	140	200
2	G_2_	40	0.3	0.01	20	55	80	0.6	0.02	55	80
